# Lipid-Polymer Hybrid Nanoparticles as a Smart Drug Delivery System for Peptide/Protein Delivery

**DOI:** 10.3390/pharmaceutics17060797

**Published:** 2025-06-19

**Authors:** Alharith A. A. Hassan, Eslam Ramadan, Katalin Kristó, Géza Regdon, Tamás Sovány

**Affiliations:** 1 Institute of Pharmaceutical Technology and Regulatory Affairs, University of Szeged, Eötvös u. 6, H-6720 Szeged, Hungary; harithth1983@gmail.com (A.A.A.H.); eslam.ramdan@mu.edu.eg (E.R.); kristo.katalin@szte.hu (K.K.); regdon.geza.laszlo@szte.hu (G.R.J.); 2 Department of Pharmaceutics, Faculty of Pharmacy, University of Khartoum, Khartoum P.O. Box 1996, Sudan; 3 Department of Pharmaceutics, Faculty of Pharmacy, Minia University, Minia 61519, Egypt

**Keywords:** emulsification methods, lipid-based nanoparticles, LPHNs, microfluidics, peptide/protein delivery, polymeric nanoparticles, SESE, DESE

## Abstract

The efficient oral delivery of therapeutic proteins and peptides poses a tremendous challenge due to their inherent instability, large molecular size, and susceptibility to enzymatic degradation. Several nanocarrier systems, such as liposomes, solid lipid nanoparticles, and polymeric nanoparticles, have been explored to overcome these problems. Liposomes and other lipid-based nanocarriers show excellent biocompatibility and the ability to encapsulate hydrophobic and hydrophilic drugs; however, they often suffer from poor structural stability, premature leakage of the loaded drugs, and poor encapsulation efficiency for macromolecular peptides and proteins. On the other hand, polymeric nanoparticles are more stable and allow better control over drug release; nevertheless, they usually lack the necessary biocompatibility and cellular uptake efficiency. Recently, lipid-polymer hybrid nanoparticles (LPHNs) have emerged as an advanced solution combining the structural stability of polymers and the biocompatibility and surface functionalities of lipids to enhance the controlled release, stability, and bioavailability of protein and peptide drugs. In this review, an attempt was made to set a clear definition of the LPHNs and extend the concept and area, so to our knowledge, this is the first review that highlights six categories of the LPHNs based on their anatomy. Moreover, this review offers a detailed analysis of LPHN preparation methods, including conventional and nonconventional one-step and two-step processes, nanoprecipitation, microfluidic mixing, and emulsification methods. Moreover, the material attributes and critical process parameters affecting the output of the preparation methods were illustrated with supporting examples to enable researchers to select the suitable preparation method, excipients, and parameters to be manipulated to get the LPHNs with the predetermined quality. The number of reviews focusing on the formulation of peptide/protein pharmaceutics usually focus on a specific drug like insulin. To our knowledge, this is the first review that generally discusses LPHN-based delivery of biopharmaceuticals. by discussing representative examples of previous reports comparing them to a variety of nanocarrier systems to show the potentiality of the LPHNs to deliver peptides and proteins. Moreover, some ideas and suggestions were proposed by the authors to tackle some of the shortcomings highlighted in these studies. By presenting this comprehensive overview of LPHN preparation strategies and critically analyzing literature studies on this topic and pointing out their strong and weak points, this review has shown the gaps and enlightened avenues for future research.

## 1. Introduction

Pharmaceutical nanotechnology is a wide umbrella that encompasses multifaceted delivery systems ranging from 10 nm to less than 1 µm and more conveniently from 50 to 500 nm for delivery of active pharmaceutical ingredients (APIs) [1,2]. These nanocarriers can help to enhance the bioavailability of poorly available APIs by improving their dissolution rate and intestinal absorption and prolonging their systemic circulation [3,4,5,6,7]. Numerous categories and classes of nanoparticles have appeared in the literature. They could be classified as nanospheres and nanocapsules based on the distribution of the API in the delivery system [8]. However, based on their structural components and other physicochemical characteristics, nanoparticles are more commonly categorized as micelles, liposomes, lipid nanoemulsions, solid lipid nanoparticles (SLNs), nanostructured lipid carriers (NLCs), lipid nanoparticles (LNPs), spongosomes, cubosomes, dendrimers, spanlastics, bilosomes, polymeric nanoparticles (PNs), nanotubes, metal nanoparticles, and lipid-polymer hybrid nanoparticles (LPHNs) [9,10,11,12,13,14,15].

Lipid-based nanocarriers such as liposomes, SLNs, NLCs, and LNPs have been extensively studied as candidates for the delivery of biopharmaceuticals. Their biodegradability, biocompatibility, and improved cellular uptake supported their application for the delivery of hydrophilic and lipophilic macromolecular drugs [5,10,16,17,18,19,20,21]. However, they still face several challenges. For instance, liposomes and LNPs have low encapsulation efficiency for very hydrophobic and macromolecular drugs, while SLNs and NLCs cannot encapsulate hydrophilic macromolecules. In addition, burst release, drug leakage, and instability issues can be attributed to all lipid-based carriers [5,21,22,23,24,25,26]. These are limiting factors that prevent their widespread application in the oral delivery of peptide/protein biopharmaceuticals. On the other hand, biodegradable polymeric nanoparticles have a high loading capacity for hydrophobic drugs and controlled drug release profiles that can be tailored by the selection of a particular polymer [10,12,27,28]. They can be prepared by relatively simple and scalable methods, and the rigid polymer matrix grants them greater stability compared to lipid-based carriers [20,29]. Several natural and synthetic polymers have been employed to prepare such carriers, and some of them, for example, poly(lactic-co-glycolic acid) (PLGA), polylactic acid (PLA), and poly(caprolactone) (PCL), have been introduced in some products approved by the FDA [30,31,32,33]. However, these polymeric nanocarriers also have demonstrated some limitations, such as polymer degradation, moderate circulation half-life, poor entrapment of hydrophilic molecules, potential biocompatibility concerns, and the utilization of toxic organic solvents in their preparation [19,25,34].

In recent decades, tremendous efforts have been made to eliminate or mitigate the intrinsic shortcomings of polymeric and lipid-based nanoparticles and promote their positive attributes, performance, and stability. One of the novel ideas is to assemble polymeric and lipid-based nanoparticles in one delivery system known as LPHNs or, alternatively, LipoParticles [12,25,28,35,36,37,38]. Such a hybrid approach aims to combine the advantages of both lipid and polymeric nanoparticles, hence offering a versatile and tunable platform for the delivery of a broad range of therapeutics, including peptide and protein drugs. Notably, LPHNs have demonstrated exceptional potential for delivery of biologics, particularly in the oral delivery of insulin [39,40,41].

Recent advances in LPHN design and the development of new materials and preparation techniques have expanded their potential for efficient delivery of macromolecular drugs. New polymer materials such as poly(β-amino esters) (PBAEs), poly(ethylene glycol)-block-poly(lactic acid) (PEG-b-PLA), poly(ethylene glycol)-block-poly(ε-caprolactone) (PEG-b-PCL), and modified chitosan derivatives have been engineered to enhance the encapsulation efficiency and stability of hydrophilic macromolecules such as peptides, proteins, and nucleic acids [42,43,44,45,46,47,48]. The availability of different types of polymers with a wide range of physicochemical properties offers greater flexibility in the rational design of the LPHN core–shell structure based on the properties of the encapsulated biopharmaceutical agent, allowing controlled nanoparticle formation and higher encapsulation efficiency. Moreover, surface modification with hydrophilic polymers and incorporation of permeation enhancers and/or P-glycoprotein (P-gp) inhibitors significantly improved transepithelial transport and bioavailability, especially in oral and mucosal administration routes [49,50,51,52]. Meanwhile, the continuous advancement in preparation methodology, such as microfluidic mixing methods, enabled greater control of particle size, uniformity, and batch-to-batch consistency and thus can enable scalable production [53,54]. Taken together, these advancements place LPHNs as a promising platform for the delivery of peptide/protein therapeutics.

Despite numerous studies reporting enhancement in oral bioavailability of peptide/protein drugs using LPHNs, the overall extent of absorption and absolute bioavailability is still suboptimal (less than 10% in most cases) and in need of significant improvements [40,55]. Each year, an increasing number of studies investigating the potential of LPHNs in peptide and protein delivery are being published, indicating a growing field of research. Therefore, a comprehensive review article summarizing and critically analyzing the existing literature on LPHNs for peptide and protein delivery would be highly valuable.

In this review, an attempt was made to set a clear definition of the LPHNs and extend the concept and area. This review will thoroughly discuss the conceiving of different types of LPHNs, firstly indicating six categories, to deliver biopharmaceuticals. Conventional and state-of-the-art preparation strategies (nanoprecipitation, microfluidic mixing, and emulsification methods) and the materials used will be shown along with representative examples. Material attributes and critical process parameters affecting the output of the preparation methods were illustrated with supporting examples to enable researchers to select the suitable preparation method, excipients, and parameters to be manipulated to get the LPHNs with the predetermined quality.

In addition, therapeutic applications through different routes of administration will be discussed. Finally, some gaps and challenges that offer potential opportunities for future studies will be highlighted. By structuring this knowledge into a unified framework, this review will serve as a foundational reference and practical guide for researchers and developers seeking to optimize LPHNs for the delivery of macromolecular therapeutics.

## 2. Methodology

A thorough literature search was conducted to identify relevant peer-reviewed articles focusing on LPHNs as delivery systems for peptide and protein therapeutics. The search was performed on different electronic databases, namely ScienceDirect, PubMed, Google Scholar, and Web of Science. The search strategy employed a combination of keywords and topic-specific terms such as “lipid-polymer hybrid nanoparticles”, “polymer-lipid hybrid nanoparticles”, “hybrid nanoparticles”, “lipomers”, “LPHNPs”, “peptide”, “protein”, “insulin”, and “lysozyme”, using Boolean operators to refine the results. The literature search covered publications until 31 December 2024. In addition, reference lists of relevant articles and reviews were also screened to identify additional studies.

**Figure 1:**
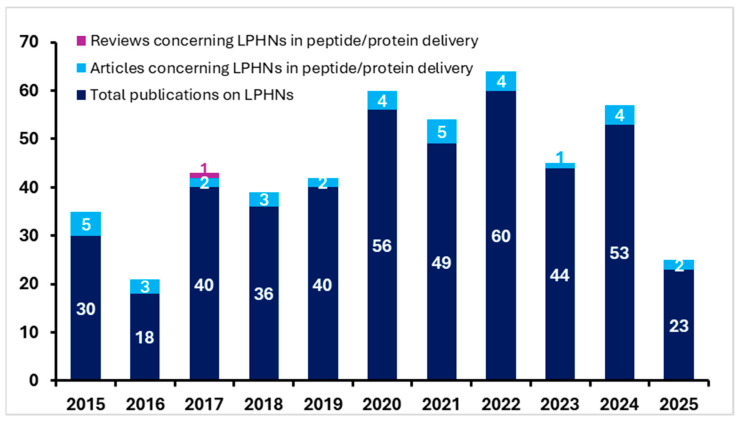
Literature analysis of publications concerning LPHNs in the past 10 years.

The retrieved articles were then manually screened and included if they met the following inclusion criteria: (1) The article was published in a peer-reviewed journal in English language and (2) the article focused on LPHNs and the loaded drug was exclusively a peptide or protein drug. Studies about (1) non-hybrid nanosystems (e.g., solely lipidic or polymeric carriers), (2) hybrid systems other than lipid-polymer (e.g., polymer-inorganic NPs or protein-polymer hybrids), and (3) non-peptide or protein-type loadings (e.g., nucleic acids, small molecules, or anticancer drugs) were used only for comparative reasons. The notable increase in the number of publications and scientific studies over the past decade (from 1 January 2015 to 1 May 2025) well demonstrates the importance of this field (Figure 1).

## 3. Lipid-Polymer Hybrid Nanoparticles

LPHN delivery systems have been extensively studied and recognized as distinguished and promising new members of the nanotechnological drug delivery systems and demonstrated manifold advantageous features. LPHNs are capable of being loaded with both water-soluble and water-insoluble materials with high entrapment yields [6,21,56,57]. They also offered enhanced capability in combinatorial drug delivery through a variety of preparation strategies, for example, in cancer chemotherapy [38,58,59,60]. In addition, the pharmacokinetic behavior of such carriers and, consequently, of the loaded API could easily be manipulated by fine-tuning their physicochemical properties [7,61]. Furthermore, controlled release of a single API and multiple APIs in a step-wise manner was successfully achieved with these carriers [13,62,63]. Moreover, functionalizing the surface of these nanocarriers with a variety of targeting compounds enabled them to carry their cargo with high efficiency to the targeted tissue/s [24,64,65,66]. Compared to polymeric and lipid-based nanoparticles, LPHNs offer multiple advantages for peptide/protein delivery (Table 1). They govern a better stability profile against enzymatic degradation and demonstrate better stability both in vitro and in vivo with high structural integrity and highly controlled drug release ability, which are mainly attributed to their polymeric component [23,61,64]. For example, coating lipid-based nanoparticles with hydrophilic polymers improves the transmucosal delivery of the carrier and consequently of the entrapped API, which can be justified partly by the stability of the transporting system in contact with the mucosal membrane brought by the polymer component and also by the enhanced interaction of the polymeric component with the mucosal layer leading to prolonged absorption time [5,16,25]. On the other hand, the lipid component boosts the advantages of these hybrid particles by conferring higher biocompatibility, biomimetic properties, and pharmacological availability. In addition, when it is employed as an outer corona in the core–shell structure, the hydrophobic lipid layer acts as a fence against the leakage of entrapped drugs by decreasing the overall exchange of water molecules with the core and consequently reducing the diffusion rate and thus promoting the API entrapment and controlling the rate of release. Moreover, this molecular fence helps in creating a sustained release profile of the entrapped API by slowing down the degradation rate of the polymer due to limited inward water diffusion [3,25,26].

However, there is no consensus on the definition of the identity of LPHNs. Some authors define this type of nanoparticle as liposomes coated by a polymeric moiety. Also, they have been recognized as a polymer shielded with mono- or multiple layers of lipid in a core–shell structure. Both definitions appear to limit the scope of this class of nanocarriers, and this umbrella remains much wider than that [13,20,67,68]. In this review, an attempt was made to further extend the concept and area occupied by the hybrid nanocarriers to enclose a variety of the studied combinations. Therefore, LPHNs could be seen as a variety of conjugations between lipid-based carriers with polymeric excipients or carriers. These conjugations could be achieved through chemical covalent bonds or noncovalent intermolecular forces such as hydrogen bonding, electrostatic interactions, van der Waals forces, and/or hydrophobic interactions [6,37,69,70]. Since the former approach leads to a new compound that needs a complete regulatory process for clinical translation [71,72], it is less preferable from a commercial aspect. Thus, in this review, the focus will be devoted more to the latter approach.

**Figure 2:**
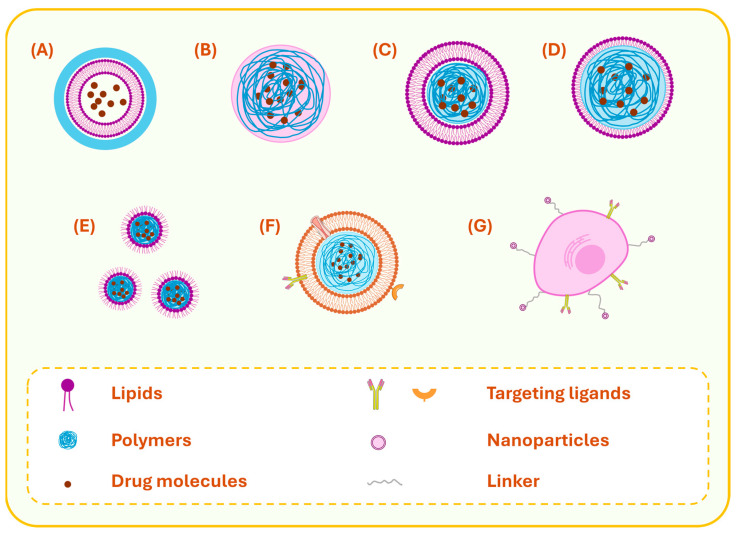
Schematic illustration of different types of LPHNs. (**A**) Lipid-core polymer-shell type; (**B**) Matrix structure LPHNs; (**C**,**D**) polymer-core lipid-shell type; (**E**) self-emulsifying LPHNs; (**F**) cell membrane bio-functionalized nanoparticles; (**G**) cell-polymeric nanoparticle hybrid vectors.

As the members of the LPHNs are continuously increasing with multifaceted structures and biological performance, the classification of such nanocarriers into sub-categories would be crucial. Based on their anatomy, LPHNs could be split into four main sub-groups, namely lipid-core polymer-shell (LC-PS), polymer-core lipid-shell (PC-LS), self-emulsifying LPHNs (SE-LPHNs), and matrix-structure LPHNs (M-LPHNs) (Figure 2A–E) [11,16,61,62,70,73]. A new generation of LPHNs called cell-based biomimetic LPHNs, which embraces cell membrane-biofunctionalized nanoparticles and cell-polymeric nanoparticle hybrid vectors, has started to present itself as a promising drug delivery platform (Figure 2F,G) [74].

LPHNs have been investigated to encapsulate a variety of APIs to treat different disorders. Among these, anticancer drugs intended for a variety of cancer cells have predominated, and most of the previous articles focused their discussions on them. This increased and excessive care is justified by the great challenges faced in this field, in particular, multi-drug resistant cancer cells [75,76,77]. It can be seen that studies on other classes of therapeutic agents such as antiepileptic, vascular system disorders, analgesics, anti-inflammatory, antibiotics, antidiabetic, and other pharmaceutically bioactive therapeutic entities, for instance, vaccines, peptides/proteins, genes, and diagnostic imaging agents, started to increasingly appear in recent years as either encapsulated, adsorbed, or covalently bonded to these nanocarriers [28,29,61,64,78,79,80].

## 4. Gastrointestinal Behavior, Oral Absorption and Cellular Uptake Mechanisms of LPHNs

LPHNs can enhance intestinal absorption of the encapsulated drugs and overcome the physiological barriers of the GI tract through different mechanisms. These benefit from the unique hybrid structure of LPHNs, offering improved pharmacokinetic behavior of the API and ultimately enhancing its stability, dissolution, absorption, bioavailability, and systemic circulation time [81].

Protection and controlled release of encapsulated drugs are primary actions by which LPHNs can improve the oral absorption of labile drugs such as peptides and proteins [5]. Encapsulating the drug within a protective matrix and minimizing its leakage from the nanocarrier is critical for drugs that can be easily affected by the strong acidic condition of the stomach or the destructive intestinal enzymes. For example, Mutlu-Agardan et al. reported that LPHN formulations based on chitosan polymeric cores and L-α-phosphatidylcholine in combination with cholesterol as lipid shells could effectively protect the loaded insulin from premature release in simulated gastric conditions and had a more efficient and prolonged blood glucose-lowering effect in a diabetic rat model compared to the polymeric nanoparticles with the same core structure [50].

Another mechanism by which LPHNs can improve intestinal absorption is mucoadhesion and enhanced mucus penetration. The presence of positively charged polymers (e.g., chitosan) on the surface of LPHNs can promote strong electrostatic interaction with the negatively charged mucus layer lining the intestinal epithelium. Such interaction prolongs the residence time of nanoparticles in the GI tract, allowing more sustained absorption and prolonged therapeutic effect [82,83]. On the other hand, mucus-penetrating LPHNs utilize a different approach to enhance oral absorption by avoiding strong adhesion to mucus and rapidly diffusing through the mucus layer to reach the absorption site at the epithelial surface. This can be achieved by coating LPHNs with a hydrophilic and neutrally charged polymer such as polyethylene glycol (PEG), which minimizes interactions with mucin and enables faster penetration to the underlying epithelium [84]. It is worth mentioning that excessive mucus adhesion may trap nanoparticles and prevent them from reaching the absorptive epithelium. Conversely, strong mucus penetration without sufficient retention may lead to rapid clearance. Therefore, keeping a balance between mucoadhesion and mucus penetration by surface optimization of LPHNs is critical for maximizing the oral absorption of these carriers [84].

**Figure 3:**
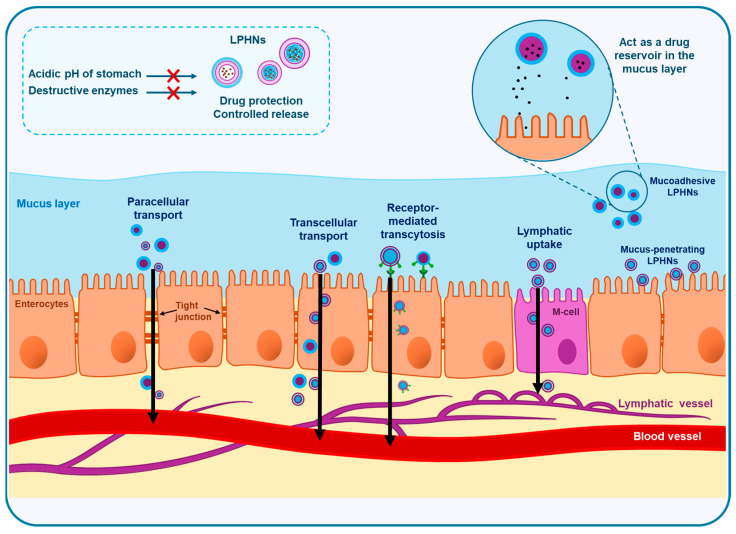
Schematic illustration showing different intestinal absorption mechanisms of LPHNs.

Once LPHNs come into contact with the intestinal epithelial surface, systemic absorption can occur through four different mechanisms depending on the physicochemical properties and surface modifications of the nanoparticles (Figure 3) [75]. The first mechanism is paracellular transport, in which LPHNs or the API released from them can cross the tight junctions between the epithelial cells [85]. In this route, particle size and surface charge of the nanoparticles are of particular importance. Smaller particles with positively charged surfaces are usually preferred. In addition, paracellular transport can be enhanced by the incorporation of permeation enhancers on the surface of LPHNs to transiently disrupt the tight junctions, allowing LPHNs or their released drugs to be freely absorbed [50,85]. The second mechanism involves transcellular transport through endocytosis, where epithelial cells internalize LPHNs through clathrin- or macropinocytosis-mediated pathways [86,87]. After internalization, nanoparticles can be trafficked across the cell and released at the basolateral membrane, enabling systemic drug delivery. Lymphatic uptake is another pathway for absorption of LPHNs, where M cells in Peyer’s patches can uptake the lipid-coated LPHNs due to their high affinity to lipophilic molecules and lipid-based nanocarriers [88,89]. After uptake by M cells, LPHNs are transported to the intestinal lymphatic system and eventually enter the bloodstream. This route allows the nanoparticles to bypass the hepatic first-pass metabolism, significantly enhancing oral bioavailability, as demonstrated with drugs like ibrutinib, where LPHNs improved bioavailability over 20-fold via lymphatic transport [89]. Lastly, ligand-conjugated LPHNs can be transported through receptor-mediated transcytosis, where surface ligands can be recognized by specific receptors on intestinal epithelial cells, promoting selective and efficient uptake [90].

An in-depth understanding of these mechanisms is critical for designing new LPHN formulations capable of traversing physical and biological barriers in the GI tract and achieving higher oral bioavailability of peptide and protein therapeutics. For example, the development of new coating materials with balanced mucoadhesion and mucus penetration properties can help LPHNs to interact with mucus without affecting their efficient penetration into the absorptive epithelial cells [82]. Concurrently, lipid shells engineered for higher affinity to M-cells can boost lymphatic uptake where LPHNs can bypass hepatic first-pass metabolism, greatly enhancing systemic delivery of the encapsulated API [88]. Moreover, incorporating permeation enhancers like sodium caprate, sodium deoxycholate, or zonula occludens toxin in the shell structure of LPHNs can transiently loosen tight junctions, facilitating paracellular transport [75,85]. Finally, controlling the physicochemical properties of LPHNs can greatly affect their intestinal absorption through paracellular and transcellular transport. Optimization of particle size and zeta potential is of particular importance, as smaller particles (<200 nm) with neutral or slightly positive surface charge showed higher intestinal absorption and significant improvement in oral bioavailability of the encapsulated drugs [91,92].

## 5. Preparation Methods

LPHNs are prepared by a variety of methods that enable the loading of a wide range of medicinal substances, irrespective of their ionicity, aqueous solubility, molecular weight, lipophilicity, and hydrophilicity [61,62,64,78]. These methods could generally be grouped into two distinct approaches; two-step methods and one-step methods (Table 2) [11,20,93]. In the two-step approach, the lipid-based nanocarriers and the polymeric nanoparticles are prepared separately and then fused in the second step [24,37,87]. On the other hand, in the single-step approach, the constituents, namely the polymer, lipid, and drug, are mixed in one pot and the self-assembled particles constitute the drug-loaded hybrid nanoparticles [19,24,87].

### 5.1. Two-Step Approach

In the early phase of LPHN preparation and development, the two-step approach was the most exploited procedure, where polymeric and/or lipid-based nanocarriers are prepared separately utilizing independent procedures. Then the carrier is combined or mixed with the counterpart carrier or excipient(s) at certain ratios in a separate new process to constitute the LPHNs. The merging or coating could be achieved by sonication, incubation with or without vortexing, or direct hydration, and that could be followed by extrusion or high-pressure homogenization for size reduction to get a more monodisperse hybrid product [17,21,37]. However, these methods are less attractive for formulators due to their technical complexity and the necessity to consider and control several variables in three separate processes (i.e., preparation of lipid and polymer carriers/components and merging) [17,24].

In this approach, conventional and nonconventional methods have been employed. Conventional processes, which involve nanoprecipitation, high-pressure homogenization, and emulsification solvent evaporation methods, are usually used on a small-scale level. If the polymeric part is used to constitute the main matrix to load the active constituent, as in the case of hydrophobic drug-loaded PC-LS LPHNs, the core is fabricated first, which consists of both polymer and API, by one of the methods mentioned so far [21,37,94]. The selection of the preparation process depends partly on the required particle size of the polymeric core and also depends on the physicochemical properties of the drug. In the case of hydrophilic APIs like biopharmaceuticals, the double emulsification solvent evaporation (DESE) method is a more suitable choice, as the API cannot be solubilized along with the polymer in the organic solvent as in the single emulsion solvent evaporation (SESE) method [21]. In both cases, high energy is introduced into the system to create the nanoemulsion, usually with the aid of emulsifiers. The evaporation of the organic solvent is achieved over several hours with the aid of stirring but could be accelerated by the application of vacuum. As a result, the polymeric nanoparticles self-assemble and precipitate and are finally collected by a suitable method. However, the resulting polymeric particles have a size of typically hundreds of nanometers and a wide particle distribution [95].

High-pressure homogenization creates polymeric nanoparticles by the formation of nano-droplets by passing through a melted polymer or polymer solution nozzle with a very narrow orifice, but it requires high-demand equipment, causing higher stress on biopharmaceuticals, and usually, the output is similar to the emulsion method in terms of particle size [95].

A third option to fabricate the polymeric nanocarriers is nanoprecipitation, which involves the mixing of a good and a poor solvent for the polymer. Upon mixing solvents by stirring, sonication, dropwise addition, or pumping into microchannels, the polymer starts to self-assemble and precipitate into nanoparticles. This technique could be exploited to produce smaller polymeric nanoparticles (sub-100 nm) compared to the SESE or DESE method with the required physicochemical properties by fine-tuning the experimental parameters such as the mixing rate, polymer concentration, and/or the type and volume ratio of the two solvents [37,95]. Nevertheless, conventional nanoprecipitation enables relatively low drug loading (<10 wt%) due to the difference in the precipitation time of the polymer/s and the drug. Drug loading may be increased by microfluidic flow-focusing, flash, sequential, or salt-induced precipitation, which can offer greater control over the precipitation time of polymers and drugs by changing salt concentrations. At the optimum salt concentration, the polymer and the drug can sequentially precipitate or co-precipitate, leading to successful encapsulation and high drug loading (up to 65 wt%) [96]. This method requires less energy, so it can be suitable for the formulation of biopharmaceuticals.

As a second step, this drug-loaded polymeric nanocarrier is merged with preformulated lipid-based nanoparticles such as liposomes or a thin lipid film by direct hydration, ultrasonication, or simple vortexing at temperatures higher than the gel-to-liquid transition temperature of the lipid component to facilitate the formation of the LPHNs due to noncovalent attraction forces [1,24,37]. As mentioned earlier, an extra step to control the particle size and the dispersity by extruding LPHN suspension through a porous membrane of a pore size equivalent to the aimed particle size or by homogenization at a temperature above the transition temperature to obtain monodisperse LPHNs might be performed [1,97].

To optimize the hybridization step to obtain LPHNs with suitable physicochemical and drug release properties and stability, the concentration of the lipid-based nanocarriers or excipient, the size homogeneity of the preformed vesicles and their surface charge, the concentration of the polymeric nanoparticles, the ionic strength of the continuous aqueous phase, and the kind and concentration of other materials used, such as the emulsifier, should be evaluated [2,17]. For example, a lipid bilayer shell will form around the polymeric core at a suitable ratio of the lipid component to the polymeric one. This shell could be changed into an irregular multilayer, and larger particles could be obtained by further increasing the concentration of the lipid component [95]. Also, it was found that the size and polydispersity of preformulated vesicles largely influence the size and homogeneity of the produced LPHNs. If PEGylation is employed in the preparation of the core–shell structures, longer PEG chains and higher concentrations of the lipid-PEG result in higher colloidal stability of the fabricated LPHNs, though a reduction in the zeta potential occurs. This is justified by the fact that steric stabilization conferred by the PEG chains is of imperative importance, making the electrostatic stabilization afforded by the lipids of secondary importance [37,98,99].

A reverse scenario could be seen when the API is contained in the lipid matrix, although this more rarely applies to biopharmaceuticals. A drug-loaded lipid-based nanocarrier can be formulated by various methods; thin-film hydration or solvent injection methods could be used in the case of liposomes, and cold or hot high-pressure homogenization or emulsion methods could be employed to prepare SLNs. Again, these lipid-based nanoparticles carrying the payload are mixed with a polymeric component to form LC-PS hybrid nanocarriers. Similarly, controlling the concentration of both components and other excipients, time, and degree of mixing will affect the output of the hybridization step [17,70,100]. For example, a two-step procedure has been employed to load salmon calcitonin peptide into LC-PS LPHNs, where the peptide-containing SLN core was prepared by the DESE method, followed by coating of the separated particles with chitosan in a separate step by simple incubation in chitosan-surfactant solution as the cationic polymer interacts electrostatically with the negatively charged lipid core and thus adsorbs on the surfaces of the SLNs. It has been found that both the concentration of the SLNs and the concentration of chitosan in the solution have affected the physicochemical properties of the LPHNs significantly. For instance, increasing the concentration of chitosan from 0.01% to 0.05% has significantly increased the zeta potential of the prepared LPHNs from approximately a neutral value up to around 26 mV. Increasing the concentration of chitosan helped to decrease the size of the nanoparticles, while increasing the concentration of the lipid particles led to a dramatic increase in the Z-average of the hybridized particles to values higher than 1 micron. Moreover, the entrapment efficiency of the peptide has apparently been affected by the concentration of the polymer [17].

On the other hand, two-step nonconventional techniques have been proposed to prepare LPHNs at higher amounts to scale up the production. These techniques involve spray drying, spray freeze-drying, and particle replication in nonwetting templates (PRINT), sometimes called soft lithography particle molding. In the PRINT technique, there is a high capability to control the shape and the size of the LPHNs with a monodisperse distribution, as it is governed by the used mold cavity [2,36].

### 5.2. Single-Step Approach

Due to the relatively long preparation time, high energy expenses, and complexity of the two-step procedures, the investigators have been looking for a relatively simple, cost-effective, scalable, and predictable alternative formulation approach. Consequently, time-saving single-step methods have emerged as attractive techniques to produce LPHNs. Currently, most of the LPHNs in the published studies have been prepared by this approach. Generally, LPHNs are prepared based on this approach either by self-assembled nanoprecipitation or modified emulsification solvent evaporation/extraction methods. These techniques are the same as mentioned so far to prepare the polymeric nanoparticles, but sometimes minor differences could be seen [7,11,62,87].

#### 5.2.1. Self-Assembly Nanoprecipitation Method

This method of preparation is suitable to prepare PC-LS-type LPHNs with a high production yield of Z-average less than 100 nm [19,56,64], but mainly loaded with hydrophobic drugs [7,26,28,101]. The general concept of this method is to dissolve polymer/s and drug/s in a water-miscible organic solvent, which is then added to the aqueous lipid dispersion in a dropwise manner to bring the lipid and polymer into close contact for self-assembly due to interactions between the hydrophobic tail of the lipids and the hydrophobic polymer core. An input energy to assist the disassembly of lipid materials and their reassembly around the polymeric cores, creating homogenous core–shell structures, is crucial and can be achieved by increasing the temperature to 60–70 °C and/or by stirring or sonication. This is usually followed by solvent evaporation and a centrifugation step to eliminate extra polymer and lipid, and then dry LPHNs could be obtained by one of the drying methods [57,61,79,102].

The lipid-to-polymer mass ratio considerably affects the quality of the produced LPHNs [20,103,104]. It was found that the ratio of lipid to polymer in a range of 10% to 20% is convenient to produce LPHNs of less than 100 nm size and surface charge between −30 and −35 mV through a single-step nanoprecipitation method [105]. Interestingly, in the following work, reducing this ratio to 1% did not influence the size stability of the hybrid particles prepared by the same method, which suggested that the entire coverage of the polymer core surface is not required to maintain such stability [57], while a high amount of lipids could result in the coexistence of large-sized vesicular structures besides LPHNs [57,105]. In addition, molecular weight, viscosity and concentration of the polymer; the type and composition of the phospholipids; the pH of the external dispersion medium; the volume ratio and miscibility of the organic and aqueous phases; the concentration of the surfactant, if used; and the concentration of the drug have apparent effects on the physicochemical properties and EE% of the produced LPHNs. Moreover, the mixing technique and the degree of mixing of the solution also have a considerable impact on the size and PDI of the hybrid particles [19,26,29,61,79]. An alternative to nanoprecipitation is ionic gelation, in which the hybrid nanoparticles are obtained upon mixing of oppositely charged lipid and polymer excipients. To achieve the required size, surface charge, and EE%, the lipid-to-polymer ratio should be optimized [35,62,106].

**Figure 4:**
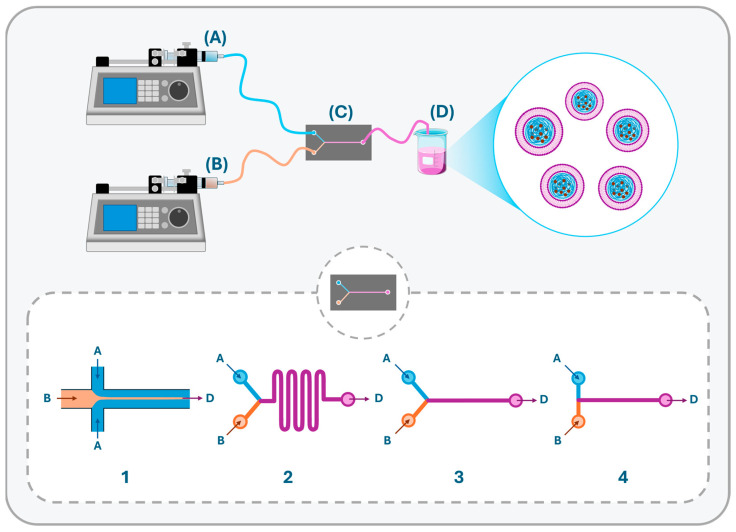
Schematic illustration of microfluidic system settings showing a variety of microfluidic devices geometries; (**A**) is an aqueous solution; (**B**) is an organic solution; (**C**) is microfluidic mixing chip; (**D**) is the formed nanoparticles output; (1) microfluidic hydrodynamic focusing (MHF); (2) Y-type with staggered herringbone micromixer (SHM); (3) Y-type without SHM; (4) T-type micromixer.

The self-assembly could be achieved as well by the nonconventional microfluidics method. This technique, which has extensively been used to formulate polymeric and lipid-based nanocarriers, also found a place to fabricate LPHNs. These miniaturized devices can manipulate small volumes of liquids in narrow channels of a few millimeters to micrometers in dimension with a greater surface area that enables effective and rapid mixing of fluids, leading to improved production yield and control over the produced particles [29,63,64]. In microfluidic chips, the mixing of molecules takes place solely through molecular diffusion, as the flow of fluids is laminar with a low Reynolds number and is governed mainly by viscous forces and pressure gradients. Consequently, a variety of geometries have been introduced, such as microfluidic hydrodynamic focusing (MHF), W-type and Y-junction with or without a staggered herringbone micromixer to further promote mixing by additional convective effects (Figure 4) [107,108], along with other alternative fluid reactors to prepare more uniform and smaller particle-size LPHNs with a higher degree of reproducibility by virtue of the enhanced vorticity and mixing efficiency [29,64]. Upon mixing of the liquid streams in microfluidic devices, the polarities change along the mixing chamber, and consequently, a nanoprecipitation reaction and rapid self-assembly of the dissolved molecules into nanostructures take place within the time scale of milliseconds. By altering the structure of chips and by controlling the process parameters, such as the flow pattern and careful manipulation of material attributes, the physical properties, drug loading capacity, and production rate of the hybrid particles can be highly fine-tuned. Thus, the microfluidic technique offers an attractive platform for industrial-scale production of LPHNs, hence facilitating industrialization and clinical translation [29,64,109,110].

Microfluidic techniques can be superior to bulk methods, since relatively higher encapsulation efficiency has been achieved with monodisperse particle distribution and more control on the particle size by modulating the flow rate, as the ratio of organic/aqueous solvent flow rate increased from 1:5 to 1:50, the particle size was reduced from 302 nm to 191 nm along with smaller PDI. The release of the entrapped lipophilic drug compared to the bulk method was also more controllable [63]. The description of the flow pattern in terms of Reynolds number is useful in explaining the degree of mixing within microfluidic devices. Reynolds numbers higher than 30 induced a rapid and strong convective profile of mixing, which led to the production of LPHNs in a size range ideal for stability and biological relevancy in a relatively high amount (3 g/h) and in a reproducible manner [111]. Therefore, the Reynolds value, along with the flow rate ratios, appears more indicative in reflecting the development of a convective pattern that is characterized by fast and effective mixing of the precursors. Additionally, the timescale of the polymer core growth (τ_grow_) seems to be critical in controlling the homogeneity of the LPHNs. Shi Xin et al. proved with computational fluid dynamics (CFD) simulation that the homogeneous assembly of the hybrid nanoparticles can only be achieved when the τ_grow_ of the polymer cores exceeds the timescale required for lipid coating (τ_coating_). Precise control of the flow pattern in terms of Reynolds number and flow rate ratio may enable the maintenance of an optimal τ_grow_ long enough to allow homogenous assembly of the LPHNs [112].

Nevertheless, it is difficult to generalize a range of Reynolds numbers required to produce LPHNs of certain quality for microfluidic devices of different geometries or for different precursors of lipids and polymers with a variety of compositions. Therefore, this represents an attractive area for research to create a roadmap for this technology to facilitate the continuous good manufacturing practice of LPHNs and facilitate clinical translation. A further problem is the limited applicability of this method for water-soluble drugs, since they are likely to leak into the aqueous medium [78]. Consequently, incorporation of biopharmaceuticals into LPHNs would not be feasible using such techniques, and this can be managed in the single-step approach by utilization of the other option, the emulsion method [2].

#### 5.2.2. Emulsification Solvent Evaporation/Extraction Method

Single-step SESE and DESE approach is suitable to load a wider range of APIs into LPHNs compared to nanoprecipitation owing to the greater flexibility [24,36,78,113], although LPHNs prepared by emulsion methods are generally larger. The selection of the approach depends mainly on the type of targeted LPHNs and the solubility of the drug [5,78]. Generally, water-soluble molecules are prepared by DESE, while insoluble ones are usually encapsulated into LPHNs by SESE [5,13,16,36].

By default, water-immiscible solvents, such as dichloromethane (DCM) since it can be removed completely because of its low boiling point and high vaporization rate [5,114,115]. are used as an oil phase in which the lipid, polymer, and drug are dissolved together. Then, it is emulsified in an aqueous phase under probe or ultra-sonication to form an oil-in-water (O/W) nano-emulsion in which hybrid particles of the lipid-coated polymeric core are formed with the hydrophobic tails of the lipids self-assembled and attached onto the polymeric core while the hydrophilic heads orient themselves towards the aqueous external phase. Subsequently, the organic solvent is evaporated to get a suspension of nanoparticles. The dry powder could be prepared by freeze-drying for extended periods of storage [2,24,116].

**Figure 5:**
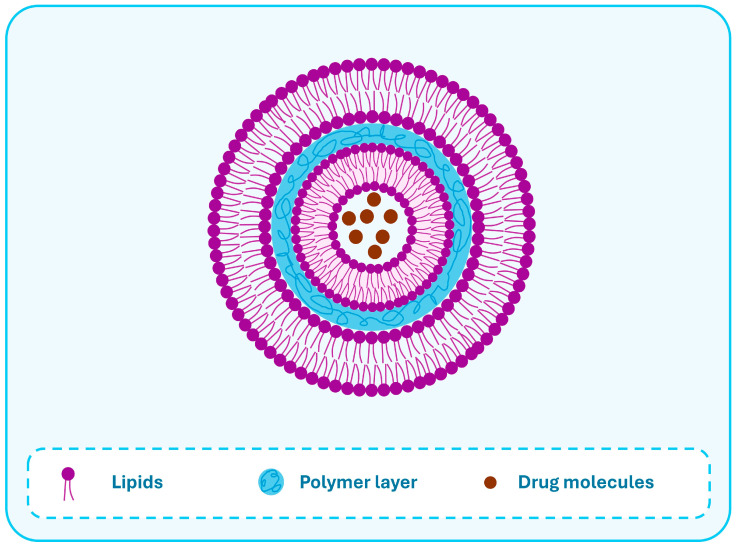
Schematic illustration of the structure of the hollow core/shell lipid-polymer-lipid hybrid nanoparticle.

In the case of water-soluble drugs, DESE is used to prepare the LPHNs [78]. The drug is dissolved in a separate aqueous phase constituting the internal phase (W_1_) and is emulsified in an oil phase containing the polymer and lipid to form a W_1_/O nano-emulsion, which is finally poured into an external aqueous phase and similarly probed or ultra-sonicated to form the double nano-emulsion (W_1_/O/W_2_). Following the evaporation of the organic phase, the formed LPHNs could be separated from other components by centrifugation. The same procedure could be followed to get the dry LPHNs as mentioned for the SESE method [5,13,36,117]. With this technique, LPHNs with special core/shell-type lipid-polymer-lipid architecture can be prepared composed of a polymer layer sandwiched between two lipid layers, and the hydrophilic active agent is contained within an aqueous core engulfed by the internal lipid layer (Figure 5) [118].

Emulsification methods are the most commonly used to incorporate hydrophilic drugs such as peptides/proteins into LC-PS and PC-LS type LPHNs for a variety of routes of administration [16,39,115,119]. For example, insulin has been loaded into hybrid nanoparticles for oral delivery using DESE [39,120]. However, using the emulsification method to prepare such drug delivery systems for peptides might raise stability issues due to:

(1) The exposure of the protein to the hydrophobic organic solvent (e.g., DCM) in the primary emulsion as the maximum degree of deactivation occurs at the organic–aqueous interface.
(2) Dehydration during the lyophilization process, which could be used to obtain a dry powder, might cause the denaturation of proteins as well.

Therefore, careful selection of lipids, polymers, solvents, and other excipients is of high importance to avoid such stability problems. Using stabilizers in the internal aqueous phase is important in protecting protein from the deactivating effect taking place at the very first step of nanoparticle formation [121]. Several stabilizers, such as serum albumins, can concurrently shield the protein from the organic solvent and also stabilize the primary emulsion by preferential accumulation at the aqueous–organic interface, thus preventing the denaturation of proteins by the organic phase. As an additional benefit, serum albumins can promote the encapsulation efficiency of proteins. Using lyoprotectant agents in the formulation of peptide-loaded LPHNs by the DESE technique helps to retain the bioactivity of proteins by reducing their aggregation during the freeze-drying process [122].

Several experimental parameters of the emulsification evaporation methods have been investigated by different research groups. These include:

1. Organic phase composition.
2. Volume of the external aqueous phase and its pH.
3. Polymer type and concentration.
4. Type of lipid or lipid combination and their ratios.
5. Concentration of lipid/s in the organic phase.
6. Polymer/lipid ratio.
7. Type of the stabilizer and its concentration.

Manipulation of these factors was done to formulate LPHNs of the required physicochemical and drug release properties with high encapsulation and loading capacity and enhanced production yield [13,41,114,121,123].

The increasing polarity and miscibility of the organic phase increases the size of the produced particles, as the use of acetone resulted in particles with the highest size of around 800 nm, while ethyl acetate and DCM gave smaller particles with a mean diameter of 225 nm and 243 nm, respectively, which can be due to the partial miscibility of acetone and water leading to its diffusion into the external aqueous phase, causing destabilization of the emulsion with bigger internal droplets [16]. However, in another work, controversial results were obtained as DCM resulted in slightly smaller particles of 309 nm compared to 334 nm obtained with the use of ethyl acetate, which has approximately 5 times higher aqueous solubility. The EE% of insulin in M-LHPNs was about 10% higher with DCM due to accelerated polymer precipitation resulting from the higher evaporation rate of the low boiling point DCM (40.1 °C) compared to ethyl acetate (77.1 °C). Interestingly, the addition of acetone in a 1/3 ratio has increased EE by about 3% and reduced the size of LPHNs by around 100 nm in the case of both individual solvents. This has been justified by the rapid diffusion of the acetone into the external aqueous phase, leading to a decrease in the interfacial tension and faster polymer precipitation with the limited escape of the encapsulated protein [41]. Nevertheless, the use of less harmful organic solvents is recommended for the production of LPHNs for protein delivery since ethyl acetate and acetone were shown to be more delicate with such biological molecules compared to DCM [114].

Emulsifiers have different effects on the physical properties of the LPHNs; they can stabilize nanoemulsions to enable minimum droplet size and consequently small and monodisperse nanoparticles [16,39,41,78,120,121,124], preserving the native structure of proteins and protecting them from the disrupting effects of the organic solvents [121], and improve encapsulation efficiency through creating complexes with peptides and proteins and so enhancing their affinity and solubility in the lipids and polymer components. For instance, the hydrophobic ion-pairing (HIP) approach in which a peptide is complexed reversibly with an oppositely charged surfactant can be used to promote the liposolubility of the hydrophilic molecule in hydrophobic core polymers reducing the partitioning of the protein into the external aqueous phase during the encapsulation process and thus enhancing the loading capacity [41,120,121,125], and also enabling higher permeation across cell membranes due to the higher lipid solubility and imparting conformational stability to the peptide against the disruptive effect of the organic solvents [125]. Nevertheless, it is not easy to generalize the relationship between the type of stabilizer, the stability of the emulsion, and the characteristics of the produced LPHNs, and more systematic experimental work is required to understand the mutual interactions with other factors.

EE also can be controlled with the pH of the aqueous phase, since bringing it near the isoelectric point (PI) of a protein could highly improve EE while keeping its native form. In a study that used lysozyme as a model peptide, a variety of formulation parameters using the single-step DESE method were investigated. The use of the anionic surfactant sodium laureth sulfate (SLS) in the internal aqueous phase gave a more stable primary emulsion and consequently smaller LPHNs and caused a considerable increase in the EE% by forming a HIP complex with lysozyme, resulting in a rise in the liposolubility of the peptide and hence more entrapment in the hydrophobic polymer compared to the non-ionic surfactant Tween 80. Higher amounts of the polymer in the organic phase led to an apparent increase in the mean particle size, and that was justified by a rise in the viscosity of the organic phase leading to larger droplets and consequently larger particles. Increasing the ratios of PC to lipid phase led to a significant increase in the EE and negativity of the surface charge without a notable change in the size of the hybrid particles [79,121]. Increasing this ratio generally decreases the size of the produced LPHNs with a single lipid bilayer coat up to a certain value, beyond which further increase may lead to either a multi-lamellar lipid shell or coexistence of other lipid carriers such as liposomes. This change in particle size in response to a change in lipid/polymer ratio is an inverse relationship with the EE% [2]. The osmotic pressure gradient between the internal and external aqueous phases can also influence EE. For example, a two times higher EE% of antigenic peptides was achieved when the osmotic pressure gradient was optimized by adding 15% glucose in the external aqueous phase [34].

Cui et al. prepared insulin-loaded M-LPHNs by a modified version of the DESE method. A complex between insulin and soybean phosphatidylcholine (SPC) was prepared first, and upon addition of the organic solvent containing a polymer, spontaneous formation of reverse micelles took place, replacing the first step of preparation of the primary emulsion. The polymer of choice was the low-Mw PLGA (Mw 9500), as it gave a higher EE%, smaller particle size, and higher zeta potential. The polymer/lipid ratio at 5:1 and the use of DCM as an organic solvent have lowered the particle size and boosted the EE% up to 90% [41].

Besides formulation parameters, sonication time may be an important independent factor of the homogenization of the primary and secondary emulsion [17,34,36]. In the case of insulin- and lysozyme-loaded LPHNs, increasing the sonication period from 60 s to 90 s, or from 2 min to 5 min, respectively, has led to more homogenous particle size distribution 128]; nevertheless, high shear forces applied for a longer period could damage the integrity of peptides [41]. Therefore, optimization of this factor is crucial, and a trade-off must be selected between the homogeneity of the hybrid system and the stability of the labile drugs. Although the emulsification method has several advantages, the residual solvent/s constitutes a major drawback that could be a cause of toxicological issues [94].

## 6. Examples of Peptides/Proteins Formulated in LPHNs

In this section, different examples of peptides and proteins encapsulated in LPHNs are discussed along with the intended route of administration (Table 3). The results of these studies focused on the capacity of drug loading and release kinetics of the LPHNs. Biocompatibility of drug-free nanocarriers, in vitro cytotoxicity of drug-loaded LPHNs, and cellular uptake have been extensively studied as well. Some authors dived further in and did in vivo studies on some experimental animal models. As mentioned earlier, the parenteral route is currently the most predominant route of administration of such therapeutic agents, and due to its drawbacks, several researchers looked for alternative, less invasive or non-invasive routes of administration using the LPHNs platform. Among these routes, the oral route is highlighted as it is the most suitable one based on patient acceptability. Additionally, manufacturing of oral formulations does not require a sterile environment, and consequently, less production cost is attained compared to injectable formulations [1,41]. Consequently, most of the mentioned studies are oriented towards this administration route.

Insulin is one of the commonly studied proteins to be delivered through an alternative non-parenteral route of administration using the LPHNs delivery platform. These studies addressed the challenges accompanying the oral delivery of such protein, including the difficulty of this large hydrophilic molecule to cross the intestinal mucosa, the unfavorable conditions of the GIT (e.g., low pH of the stomach and extensive activity of proteolytic enzymes), and the low encapsulation efficiency in other delivery platforms.

M. Boushra et al. (2016) formulated M-LPHNs for oral delivery of insulin in two separate studies [39]. In both of them, they used the same lipid components and preparation procedure, but they changed the polymer type from methyl cellulose in one study to propylene glycol (PG) in the other one. In both cases, the EE% has been promoted dramatically compared to the SLN counterpart by the creation of hydrophilic regions within the lipid matrix and stabilization of the primary emulsion attributed to the surface-active properties of methylcellulose. PG improved the entrapment by enhancing the viscosity of the internal aqueous phase and initiating H-bonding with the protein, leading to inhibition of the migration of insulin to the water/organic solvent interface. Both LPHN systems showed good dispersion stability at gastric and intestinal pHs as well as storage stability. Moreover, these nanocarriers showed extensive uptake by Caco-2 cells, indicating a high potentiality of absorption on oral administration. However, LPHNs formulated with PG showed superiority over those with methocel in terms of EE%, smaller particle size, and lower PDI [39,127]. Unfortunately, the pharmacological bioavailability would not be available for comparison due to the lack of in vivo study in the case of PG-containing LPHNs.

Cui et al. used an opposite principle to the previous work. They enhanced the lipophilicity of insulin to increase its entrapment in the hydrophobic carrier. A complex of SPC and insulin prepared by the co-solvent lyophilization technique was loaded into hydrophobic polyester polymers using the emulsification solvent evaporation method and resulted in M-LPHNs type composed of insulin-containing reverse micelles embedded into the polymeric nanoparticles. Through manipulating different experimental parameters, monodispersed hybrid nanoparticles of approximately 200 nm size and high encapsulation of insulin of about 90% were produced, and their relative pharmacological bioavailability was investigated in vivo on diabetic rats [41]. Although they achieved a higher relative bioavailability of 7.7% compared to the M-LPHNs of Boushra et al. (2016), this value remains much lower than that required in clinical situations (>15%) [39]. This hybrid system seems promising in oral delivery of insulin, and by surface modification approaches and inclusion of some excipients such as enzyme inhibitors, this relative bioavailability could be highly pushed towards the required values. However, this study, despite its strong points of in vitro release and in vivo behavior investigations, lacked important testing of the storage and colloidal stability of such nanoparticles in simulated gastric and intestinal fluids.

M. García-Díaz et al. followed the strategy of the previous study to enhance the EE% of orally administered insulin-loaded hybrid nanoparticles [120]. Similarly, insulin-lipid complexes with SPC or sodium caprate (NaC_10_) were formulated to increase the liposolubility of insulin in the hydrophobic polyester PLGA. They followed the general procedure of the DESE technique without the modifications used in the previous study (reverse micelles-solvent evaporation). In both types of complexes, comparable monodispersed hybrid nanoparticles were obtained to the previous work in terms of size and EE% [120]. The apparent difference was the surface charge of the particles, which was higher towards the negative values, approx. −30 vs. –17 mV, respectively. Also, the in vitro release study showed a higher burst release pattern with a higher proportion of free insulin in a simulated intestinal fluid. Almost 80% of the loaded insulin was released within only 1 h in comparison to 24 h needed to give a similar percentage in the previous work. Both observations might be due to the higher proportion of surface-entrapped insulin-lipid and also reflect the weaker association of the lipid and the drug in the complexes. This could be highly attributed to the variation in the preparation technique that led to this difference in the release profile.

B. Sarmento et al. (2011) developed insulin-loaded LC-PS hybrid nanoparticles prepared in two steps, including the DESE method for Witepsol-based SLN followed by coating with the cationic polysaccharide chitosan under stirring [126]. These nanoparticles were proved to evade the opsonization and phagocytosis by the mononuclear phagocyte system (MPS) by using an in vitro macrophage cell line. This study shows the superiority of the hybrid platform over the SLN in elongation of the blood circulation of the intestinally absorbed nanoparticles benefiting from the surface characteristics imparted by chitosan that rendered the surface more hydrophilic and switched the surface potential from negative to positive values. These changes made the nanoparticles much less efficiently phagocytosed, as opsonins prefer hydrophobic, negatively charged nanoparticles to adsorb on [126]. The EE% (between 39% and 66%) and loading capacity (0.3–0.5%) of insulin reported by the authors were significantly lower than other developed M-LPHN types for oral insulin delivery [41,120]. Therefore, this core–shell hybrid system needs further enhancement to be clinically feasible, in addition to its advantage of enhanced bioavailability and stability of the loaded peptide, in terms of drug loading efficiency.

A closely related work on oral insulin delivery was done by P. Fonte et al. using the same preparation technique and polymer and lipid components to formulate LC-PS-type LPHNs. The potentiality of this study comes not just from the in vitro absorption experiment but also from the in vivo study done on diabetic rats. This hybrid nanoparticle system showed a superior ability to stabilize and protect the encapsulated protein over the non-hybrid SLN, as well as a more sustained hypoglycemic effect, and achieved promising relative pharmacological bioavailability (≈17%), which was double that of the uncoated SLN (≈8%), thanks to enhanced paracellular permeability and the mucoadhesive property of the polymer [16]. This relative bioavailability is among the highest values reported for oral insulin delivery entrapped in a polymeric or lipid-based system or even LPHNs, making this system a potential candidate for clinical translation. However, this investigation lacked an important aspect of measuring the storage stability of those formulated LPHNs, especially with the relatively large size and wide PDI values reported (Table 3).

Earlier work by M. García-Fuentes et al. showed the improved oral delivery of insulin through the merging of a lipid carrier with a polymer in the form of LC-PS nanoparticles. They focused on the comparison of the stability of different prepared formulations and found that hybrid nanoparticles with PEG showed the highest stability both in simulated gastric and intestinal fluid, as the particle size remained the same and the molecular degradation of the lipid core was less than 5% in the gastric medium. On the other hand, the non-hybrid formulation (SLN) showed considerable and immediate aggregation in the gastric medium to a degree that the assay of the remaining amount of the lipid could not be done, although their absolute zeta potential value was higher than that of the PEGylated particles (−33 mV vs −20 mV). This agrees with other reports that demonstrated that steric stabilization is more important than electrostatic repulsions in the colloidal stability of LPHNs [7,19,37]. Although nanoparticles with a PEG-2000 outer shell were selected to load insulin, the protection action was the same for both grades of the PEG and was attributed to its steric stabilizing effect, which is not compromised by the low pH condition of the stomach, and additionally the impaired attachment of the digestive enzymes by virtue of the well-known protein-rejecting characteristics of this polymer [23]. However, EE% of the drug was not reported and the release profile in an in vitro setting was not optimum to get a clear conclusion. Moreover, no in vivo studies have been done to show the efficacy of these proposed LPHNs.

Hybrid nanocarriers have also shown marked superiority, based on their advantages, in delivering APIs to treat brain disorders via noninvasive intranasal administration. By this route, the drug-loaded nanocarrier can bypass the blood–brain barrier (BBB) by slow intra-axonal transport through the olfactory and trigeminal nerves or by an accelerated transfer via the perineural space surrounding the nerve cells [129]. Olanzapine has been loaded into a hybrid system of nanoemulsion coated with chitosan to be delivered through the nose-to-brain pathway. It has been reported that the cumulative amount and the maximum drug concentration achieved in rat brain were much higher than the non-hybrid counterpart [130].

An example of a peptide delivered by LPHNs via this route to treat a brain disorder is the neurotrophic factor human insulin-like growth factor-I (hIGF-I). By this route, the rapid degradation rate, fast clearance of the peptide from the systemic circulation, and limited ability to pass the BBB by virtue of its high Mw and hydrophilicity could be overcome. LC-PS nanoparticles comprising NLCs as a core and chitosan as a polymer shell were formulated. This hybridization offered a solution for the major shortcomings of the non-hybrid NLCs after intranasal application, which are a short residence time in the nasal cavity and incomplete medicine absorption because of the mucociliary clearance. In addition to the suitable size, PDI, and enough colloidal stability of the LPHNs, the EE% value was quite high, approximately 90% [119]. Benefiting from the properties of the polymer shell, these positively charged nanoparticles are speculated to be highly attracted to the negatively charged cell membrane of the nasal epithelial cells, and together with the mucoadhesive characteristics and an ability to transiently open epithelial tight junctions, these nanoparticles could highly promote the drug uptake through the nasal mucosa and access to the CNS [131]. The LPHNs loaded with a near-infrared dye were detected by the fluorescence imaging monitoring in the nasal cavity and brain of female rats up to 24 h after intranasal administration, confirming prolonged contact of these nanoparticles with the nasal epithelium and their accumulation not only in the frontal brain section but also deep penetration into, for example, the cerebrum and hippocampus segments. Moreover, these LPHNs demonstrated no signs of toxicity during both in vitro and in vivo investigations [119]. However, this work had some limitations that need to be performed or promoted in the future. For instance, the peptide-loaded LPHNs were not investigated in the experimental animals, and instead, the authors used the dye-loaded nanoparticles for in vivo drug delivery. This might be due to the difficulty of measuring the absorbed amount of the peptide in the brain tissues. Also, a higher proportion of the near-infrared dye was detected in the lung, indicating a higher accumulation of the nanoparticles in this tissue. Therefore, further modifications and optimization are needed to mitigate the delivery of the drug to other tissues rather than the brain.

LPHNs have been investigated to deliver vaccines using bovine serum albumin (BSA) as a model antigen. The hybrid particles were formulated from a polymeric core of PLGA, which encapsulated the BSA and provided rigid support for the hybrid particles, and a lipid shell containing the cationic lipid DOTAP and cholesterol PEGylated with DSPE-PEG(2000) amine. This engineered outer corona of lipid functioned as a biocompatible shield and a bi-directional reduced permeability barrier due to its content of cholesterol. This mitigates the entrance of the external water molecules or serum proteinases, thus minimizing the degradation of the PLGA core and consequently preventing rapid leakage of the loaded protein and offering the prolonged and controlled release of the antigen, thus avoiding premature degradation. One of the distinguished notes of this study is the reported high entrapment efficiency of the protein, which was at a minimum of 88.5% [67]. Unfortunately, this high percentage of the encapsulation of this hydrophilic macromolecule in the hydrophobic polymer was not justified or explained by the authors, who used a two-step method to prepare such hybrid particles. It is worth mentioning that although PEGylation has enhanced the storage stability remarkably and saved the initial physical characteristics of the LPHNs, it inversely affected the uptake of the hybrid particles by an in vitro culture of dendritic cells via endocytosis at higher PEGylation ratios, leading to undermining the required immune response triggering [67]. Therefore, the lesson learned from this work is that the suitable PEGylation ratio should be cautiously determined as a trade-off between the long-term stability and the in vivo performance of the hybrid nanocarriers, for instance, in vaccination.

Another commonly studied protein in the field of formulation development was lysozyme [22,114,125,132]. It has a number of activities, such as antibacterial, antiviral, anti-inflammatory, anticancer activity, and immunomodulation [133,134]. A group of researchers from Ankara University reported different formulations of PC-LS LPHNs loaded with lysozyme using the DESE method through a series of published papers. To get relatively stable hybrid nanoparticles with high EE% and have a suitable size and size distribution, they investigated and manipulated a variety of formulation parameters. These include total lipid concentration in the organic solvent, the relative ratios of the used lipids to each other, organic solvent composition, the concentration of the employed polymer PCL in the organic phase, volume of the external aqueous phase, and type of the stabilizer in the internal aqueous phase. It is expected that some of the enzymatic activity of the protein might be lost during the preparation of the nanoparticles, and they found that this loss came mainly from the lyophilization process and not from the DESE method [114,121]. This loss could be minimized by using a suitable cryoprotectant such as sucrose, mannitol, or trehalose [122,135]. It is worth mentioning that the observed cell viability was higher than 75% for both the nonhybrid PCL particles and the hybrid ones in the MTT assay on a cell line. However, the cell uptake of the LPHNs was significantly higher compared to PCL nanoparticles, reflecting an improved interaction of the LPHNs with cell membranes [114].

The basic peptide hormone calcitonin has been clinically used for more than 40 years in the form of salmon calcitonin to control severe hypercalcemia and for metabolic bone diseases such as Paget’s disease and osteoporosis and is currently available in the pharmaceutical market as a nasal spray and intravenous, subcutaneous, and intramuscular injectable preparations [4,136,137]. Due to its high water solubility and extreme instability in the GIT, the absorption of this peptide through the GIT mucosa is very low [4]. M. Garcia-Fuentes et al. formulated three different oral preparations of LC-PS LPHNs loaded with salmon calcitonin by changing the external polymer layer and, in one formulation, by incorporating Miglyol^®^ 812 (liquid triglyceride) in the lipid core (Table 3). Nanoparticles coated with chitosan have inverted the negative surface charge of the uncoated SLN to positive values. This inversion has been accompanied by an apparent increase in the particle size by approximately 100% to values that do not support the facilitated transportation through the mucosal membrane and long-term blood circulation [126,138], along with relatively high size distribution. Additionally, coating with chitosan has affected the EE% of the peptide inversely and reduced the entrapment efficiency to around 1/3 of the value of the nonhybrid SLNs. This has been justified by the fact that the peptide (PI = 10.4) is positively charged in the pH values of the medium and is associated with the negatively charged lipid particles mainly by the electrostatic interaction. The cationic polymer is competing with the associated salmon calcitonin molecules in their binding to the lipids and displacing part of them, leading to a reduction in the EE%. On the other hand, PEG-coated nanoparticles demonstrated suitable Z-average and PDI values with high EE% above 90%. Introducing the liquid triglyceride into the formulation has slightly reduced both the size and the PDI but did not influence the EE% of the salmon calcitonin. In the case of both polymers, in an in vitro release test, the loaded peptide has been released in a two-phase pattern: an initial burst release related to the surface-associated peptide molecules, followed by a slow and sustained release phase, and approximately 40% of the load has been released within 6 hrs. However, the incorporation of Miglyol^®^ 812 did not affect the release pattern of the drug. Chitosan-coated SLNs showed an increase in the particle size upon their incubation for 1 h in simulated gastric and intestinal fluids by about 50 nm and 100 nm, respectively [17]. Indeed, this increase could be viewed as a sign of instability and could inhibit the absorption of the nanoparticles. However, this study lacks the in vitro and in vivo evaluation of the performance of these hybrid nanoparticles.

Among the reasons mentioned earlier for formulating LPHNs is enhancing the encapsulation efficiency of the hydrophilic macromolecular drugs. Tan et al. have developed an LPHNs-vaccine formulation and utilized three antigenic peptides to evoke anti-tumor cytotoxic T lymphocyte responses (Table 3). All these peptides were loaded into PC-LS LPHNs in separate formulations. It is worth mentioning that the lipids have also worked as surfactants, eliminating the need for external ones. The EE% of three peptides, TRP2, p15E, and hgp100, was improved from 24.1%, 1.05%, and 0.4% to about 30.1%, 12.1%, and 2.3%, respectively, in comparison to the non-hybrid PLGA polymeric formulations. By an in vitro experiment, these LPHNs were successfully taken up by the dendritic cells with endosomal localization. Also, these nanocarriers showed a controlled and prolonged release pattern of the peptides and needed approximately two weeks to empty their loads. These individually fabricated peptide-loaded hybrid nanoparticles were able to induce antigen-specific T cells after intradermal administration in mice, and when mixed before vaccination of mice inoculated with B16-F10 murine melanoma cells, they revealed superiority over single peptide-loaded LPHNs in suppression of tumor growth. This strategy of the combination was found successful in reducing the tumor immune escape, for instance, by downregulation mechanisms of antigens employed for vaccination [34]. This study demonstrated another area of application of LPHNs in the delivery of peptides in prophylactic cancer immunotherapy.

## 7. Conclusions and Future Opportunities

LPHNs present themselves as a promising delivery platform, combining the merits of lipid-based and polymeric nanocarriers and mitigating their shortcomings. With six different architectures, LPHNs showed their superiority in terms of physicochemical properties, EE%, tissue and cell internalization, and in vitro and in vivo stability both for the carrier and the entrapped medicines. LPHNs demonstrated their potential to protect and deliver fragile peptide/protein drugs with a higher EE%, for instance, 10-fold compared to the nonhybrid counterparts. A variety of preparation methods have been used to prepare such nanocarriers, with an apparent trend of shifting from conventional two-step to nonconventional one-step strategies. Tremendous work and a variety of excipients, including emulsifiers and stabilizers, were exploited to enhance the encapsulation and stability of the loaded proteins in LPHNs, but still, this avenue remains open for more studies to simplify and scale up the production of these nanoparticles. Combining the emulsification methods, being the most commonly used technique to encapsulate peptide/protein drugs in LPHNs, with the nonconventional techniques such as inkjet and other printing technologies and spray drying could improve the production of biopharmaceutical-loaded LPHNs in commercial amounts, facilitating their clinical translation and marketing. Moreover, exploiting quality by design principles is highly likely to accelerate the phase of development and show a variety of material attributes, such as the type of solvents, lipids, polymers, their molecular weight and concentration, and process parameters that affect the quality of the prepared LPHNs and facilitate the formulation of solid-based conclusions for industrial and regulatory affairs [139].

Concerning formulations in dosage forms, this field of research remains promising, but several issues need to be disclosed. For example, one of the potential routes of administration of small molecular drugs as well as large molecular weight biological therapies is the buccal mucosa. A variety of nanoscale delivery platforms, such as polymeric nanoparticles, have been reported to deliver small molecules as well as macromolecular drugs through this route. To our knowledge, there is no published work that used LPHNs to deliver peptide/protein through buccal administration. However, benefiting from the potentialities of this route, these nanocarriers could constitute a highly promising candidate to deliver biopharmaceutical therapies, for example, in the form of mucoadhesive buccal films to induce both local and systemic effects [140,141,142,143,144]. It could also help in controlling and tailoring the release profile of their payloads. Moreover, LPHNs could play a significant role in overcoming the delivery of macromolecular drugs through the skin barrier, which is considered much more resistant compared to the buccal mucosa.

Although there is a considerable number of in vitro stability and release studies on peptide/protein encapsulated in LPHNs, the in vivo studies remain relatively limited. Consequently, there is an eager need for more studies to investigate the stability of these biological compounds entrapped in LPHNs through a variety of routes of administration and their pharmaceutical bioavailability in animal models. This could greatly hasten the development of this platform and potentially make the clinical translation of such nanoparticles feasible in the near future.

## Figures and Tables

**Table 1:** Comparison between lipid-based nanocarriers, polymeric nanocarriers, and LPHNs.

Feature	Lipid-Based NCs	Polymeric NCs	LPHNs
Stability	Moderate (lipid oxidation, drug leakage)	High (rigid matrix)	High (rigid polymer core)
Biocompatibility	Excellent	Moderate (depending on the polymer nature)	Excellent (lipid shields polymer)
Encapsulation	Moderate for proteins	Low for hydrophilic macromolecules	Higher for hydrophilic/hydrophobic peptides/proteins
Cellular Uptake	High (membrane fusion, endocytosis)	Variable	High (lipid-mimic + polymer protection)
Bioavailability	Limited for peptide/protein due to stability issues	Moderate due to lower transcellular absorption	Improved due to combined stability and biocompatibility
Drug Release	Fast, burst due to fragile lipid structure	Controlled, tunable	Controlled due to polymer core matrix
Preparation	Simple self-assembly	More complex	Most complex due to hybrid structure, often require multiple steps
Scalability	Established with moderate challenges	Established with moderate challenges	Still emerging with more difficult challenges

**Table 2:** Comparison between single-step and two-step approaches for preparation of LPHNs.

	Single-Step Approach	Two-Step Approach
Definition	Simultaneous formation of lipid and polymer components in one process	Separate formation of polymer core and lipid shell, followed by their combination
Complexity	Relatively simple	More complex and time-consuming
Control over structure	Limited control over core–shell architecture	Greater control over nanoparticle architecture
Encapsulation efficiency	May be lower or variable	Generally higher due to optimized core loading before lipid coating
Scalability	More scalable and suitable for industrial production	Less scalable due to complexity
Reproducibility	Higher, due to fewer steps and reduced variability	Lower, due to multiple processing stages
Stability of the API	May be compromised during the preparation due to harsh conditions and/or organic solvent exposure	Improved stability due to drug encapsulation during the polymer core formation in gentler conditions
Suitable applications	When stability of the API is not a concern	More suitable for peptide/protein encapsulation due to the less harsh conditions and/or organic solvent exposure compared to the one-step approach

**Table 3:** Examples of studies that used LPHNs to deliver protein/peptide drugs.

Type of LPHNs	Peptide/Protein	Lipid	Polymer	Preparation Method	Disease/Route	Results	Ref.
LC-PS	Insulin	Dynasan^®^ 116 + Lecithin	Cholic acid or Synperonic^®^ F68 or PEG-2000 stearate or PEG-4500 stearate	Single-step DESE	Diabetes mellitus/Oral	Ps: 198 ± 4.7 nm PDI: 0.24–0.28 Zeta P.: −19.9 ± 0.9 mV EE%: -----	[23]
M-LPHNs	Insulin	SPC	PLGA (Av.Mw 9500) or PLGA (Av.Mw 18,000) or PLA	A- Ins-SPC complex by anhydrous co-solvent lyophilization. B- Reverse-micelle + ESE	Diabetes mellitus/Oral	Ps: 201 ± 25 nm PDI:----- Zeta P.: −17.8 ± 8.6 mV EE%: 88.5 ± 9.7% RB:7.7%	[41]
LC-PC	Insulin	Witepsol 85E/or Compritol 888 ATO/or Precirol ATO 5	Low Mw Chitosan	Two-step DESE for SLN + coating by incubation under stirring	Diabetes mellitus/Oral	Ps: 191 ± 24 nm PDI:------ Zeta P.: +28.2 ± 1 mV EE%: 57.3 ± 0.2%	[126]
LC-PC	Insulin	Witepsol 85E	Low Mw Chitosan	Two-step DESE for SLN + coating by incubation under stirring	Diabetes mellitus/Oral	Ps: 470 ± 32 nm PDI: 0.63 ± 0.02 Zeta P.: +34.2 ± 3.4 mV EE%: 52.2 ± 5.3% RB.: 17.7%	[16]
M-LPHNs	Insulin	SPC or NaC 10	PLGA	A- Ins-lipid complex by self-assembly and lyophilisation. B- DESE	Diabetes mellitus/Oral	(SPC) Ps: 202 ± 10 nm PDI: 0.10 ± 0.03 Zeta P.: −30 ± 5 mV EE%: 90 ± 7%	[120]
						(NaC10)Ps: 222 ± 8 nm PDI: 0.12 ± 0.04 Zeta P.: −30 ± 2 mV EE%: 91 ± 7%	
M-LPHNs	Insulin	SPC + trimyristin	Methocel A	Single-step DESE	Diabetes mellitus/Oral	Ps: 367.43 ± 9.85 nm PDI: 0.28 ± 0.01 Zeta P.: −39.4 ± 1.44 mV EE%: 40%	[39]
M-LPHNs	Insulin	SPC + trimyristin	PEG400 or PEG600 or propylene glycol	Single-step DESE + Filtration under vacuum/freeze drying	Diabetes mellitus/Oral	Ps: 203.6 ± 21.3 nm PDI: 0.175 ± 0.029 Zeta P.: −43.3 ± 0.6 mV EE%: 54.5% RB.: 5.1%	[127]
PC-LS	Lysozyme	Tripalmitin + PC	PCL	Single-step DESE	-	Ps: 110.43 ± 1.68 nm PDI: 0.29 ± 0.024 Zeta P.: −21.77 ± 0.93 mV EE%:---------	[121]
PC-LS	Lysozyme	Tripalmitin + PC	PCL	Two-step ESE (Solid-in-oil-in-water emulsification)	-	Ps: 112.53 ± 0.133 nm PDI: 0.164 ± 0.012 Zeta P.: −2.9 ± 0.04 mV EE%: 42.85 ± 0.08%	[125]
PC-LS	Lysozyme	Tripalmitin + PC	PCL	Single-step DESE	-	Ps: 58.04 ± 1.95 nm PDI: 0.15 ± 0.015 Zeta P.: −32.91 ± 2.94 mV EE%: 55.36 ± 0.21%	[114]
LC-PS	Salmon calcitonin	Tripalmitin + Lecithin ± Miglyol 812 (In case of PEG)	PEG-2000 stearate or Chitosan	Single-step DESE (PEG)	Oral	(PEG without Miglyol) Ps: 226.4 ± 7.5 nm PDI: 0.25 ± 0.07 Zeta P.: −34.8 ± 2.8 mV EE%: ≈90%	[17]
						(PEG with Miglyol) Ps: 207.4 ± 19.1 nm PDI: 0.18 ± 0.08 Zeta P.: −36.6 ± 2.5 mV EE%: ≈90%	
				Two-steps DESE for SLN + coating by incubation (chitosan)		(Chitosan) Ps: 537.7 ± 16.6 nm PDI: 0.43 ± 0.14 Zeta P.: +29.2 ± 6.7 mV EE%: 30.7 ± 2.3%	
LC-PS	-	Tripalmitin + Lecithin + Miglyol 812	PEG40-stearate	Single-step DESE	-	Ps: 187 ± 5 nm PDI: 0.21–0.23 Zeta P:-------	[115]
PC-LS	TRP2/hgp100/p15E (peptides)	DOPC + DSPE-PEG-2000	PLGA	Single-step DESE	Cancer vaccination/intradermal	Ps: 102 ± 12 nm PDI: -------- Zeta P:------- EE%: 30.1 ± 5.1% (TRP2) EE%: 12.1 ± 2.4% (p15E) EE%: 2.3 ± 0.7% (hgp100)	[34]
PC-LS	BSA	DOTAP + cholesterol + DSPE-PEG(2000) amine	PLGA	Two-steps A- DESE + freeze drying (BSA-containing PLGA NPs) B- Lipid film hydration (Liposomes). C- Homogenization + sonication + freeze drying (LPHNs)	-	Ps: 114 nm PDI: 0.067–0.109 Zeta P.: +4.78–+5.58 mV EE%: 88.5–98.3%	[67]
LC-PS	Neurotrophic factor hIGF-I	Precirol ATO5 or Dynasan114 and Miglyol^®^	Chitosan	Two-steps A-Emulsification to form NLC B-coating by incubation + lyophilisation	Brain disorder/Brain drug delivery after intranasal administration	Ps: 114.48 ± 20.20 nm PDI: 0.287 ± 0.05 Zeta P.: +28.40 ± 2.83 mV EE%: 90.28 ± 0.4% RB:--------	[119]
PC-LS	Bromelain: combination of proteolytic enzymes	Phosphatidylcholine	PLGA	Single-step DESE + centrifugation, washing, and resuspension	Oral	Ps: 209.4 ± 7.30 nm PDI: 0.134 Zeta P.: −42.1 ± 0.55 mV EE: 83%	[128]

RB: relative bioavailability. NaC10: Sodium caprate. TRP2: H-2K^b^-restricted epitope corresponding to amino acids 180–188 of mouse (SVYDFFVWL). hgp100: H-2D^b^-restricted epitope corresponding to amino acids 25–33 of human (KVPRNQDWL). p15E: H-2K^b^-restricted epitope corresponding to amino acids 604–611 (KSPWFTTL).

## References

[1] Dave V., Tak K., Sohgaura A., Gupta A., Sadhu V., Reddy K.R. (2019). Lipid-Polymer Hybrid Nanoparticles: Synthesis Strategies and Biomedical Applications. J. Microbiol. Methods.

[2] Hadinoto K., Sundaresan A., Cheow W.S. (2013). Lipid–Polymer Hybrid Nanoparticles as a New Generation Therapeutic Delivery Platform: A Review. Eur. J. Pharm. Biopharm..

[3] Kong S.D., Sartor M., Hu C.-M.J., Zhang W., Zhang L., Jin S. (2013). Magnetic Field Activated Lipid–Polymer Hybrid Nanoparticles for Stimuli-Responsive Drug Release. Acta Biomater..

[4] Sakuma S., Suzuki N., Kikuchi H., Hiwatari K., Arikawa K., Kishida A., Akashi M. (1997). Oral Peptide Delivery Using Nanoparticles Composed of Novel Graft Copolymers Having Hydrophobic Backbone and Hydrophilic Branches. Int. J. Pharm..

[5] Tang B., Qian Y., Fang G. (2020). Development of Lipid–Polymer Hybrid Nanoparticles for Improving Oral Absorption of Enoxaparin. Pharmaceutics.

[6] Men K., Liu W., Li L., Duan X., Wang P., Gou M., Wei X., Gao X., Wang B., Du Y. (2012). Delivering Instilled Hydrophobic Drug to the Bladder by a Cationic Nanoparticle and Thermo-Sensitive Hydrogel Composite System. Nanoscale.

[7] Salvador-Morales C., Zhang L., Langer R., Farokhzad O.C. (2009). Immunocompatibility Properties of Lipid–Polymer Hybrid Nanoparticles with Heterogeneous Surface Functional Groups. Biomaterials.

[8] Wakaskar R.R. (2018). General Overview of Lipid–Polymer Hybrid Nanoparticles, Dendrimers, Micelles, Liposomes, Spongosomes and Cubosomes. J. Drug Target..

[9] Cho K., Wang X., Nie S., Chen Z., Shin D.M. (2008). Therapeutic Nanoparticles for Drug Delivery in Cancer. Clin. Cancer Res..

[10] Yasaswi P.S., Shetty K., Yadav K.S. (2021). Temozolomide Nano Enabled Medicine: Promises Made by the Nanocarriers in Glioblastoma Therapy. J. Control. Release.

[11] Bou S., Wang X., Anton N., Bouchaala R., Klymchenko A.S., Collot M. (2020). Lipid-Core/Polymer-Shell Hybrid Nanoparticles: Synthesis and Characterization by Fluorescence Labeling and Electrophoresis. Soft Matter.

[12] Hassan D., Omolo C.A., Fasiku V.O., Mocktar C., Govender T. (2020). Novel Chitosan-Based pH-Responsive Lipid-Polymer Hybrid Nanovesicles (OLA-LPHVs) for Delivery of Vancomycin against Methicillin-Resistant Staphylococcus Aureus Infections. Int. J. Biol. Macromol..

[13] Yalcin T.E., Ilbasmis-Tamer S., Takka S. (2018). Development and Characterization of Gemcitabine Hydrochloride Loaded Lipid Polymer Hybrid Nanoparticles (LPHNs) Using Central Composite Design. Int. J. Pharm..

[14] Sharma B., Chauhan I. (2024). A Review: Bilosomes as Nanocarriers. CNANOM.

[15] Kumar L., Rana R., Kukreti G., Aggarwal V., Chaurasia H., Sharma P., Jyothiraditya V. (2024). Overview of Spanlastics: A Groundbreaking Elastic Medication Delivery Device with Versatile Prospects for Administration via Various Routes. CPD.

[16] Fonte P., Nogueira T., Gehm C., Ferreira D., Sarmento B. (2011). Chitosan-Coated Solid Lipid Nanoparticles Enhance the Oral Absorption of Insulin. Drug Deliv. Transl. Res..

[17] Garciafuentes M., Torres D., Alonso M. (2005). New Surface-Modified Lipid Nanoparticles as Delivery Vehicles for Salmon Calcitonin. Int. J. Pharm..

[18] Lacatusu I., Badea N., Murariu A., Oprea O., Bojin D., Meghea A. (2013). Antioxidant Activity of Solid Lipid Nanoparticles Loaded with Umbelliferone. Soft Mater..

[19] Chan J.M., Zhang L., Yuet K.P., Liao G., Rhee J.-W., Langer R., Farokhzad O.C. (2009). PLGA–Lecithin–PEG Core–Shell Nanoparticles for Controlled Drug Delivery. Biomaterials.

[20] Tahir N., Madni A., Balasubramanian V., Rehman M., Correia A., Kashif P.M., Mäkilä E., Salonen J., Santos H.A. (2017). Development and Optimization of Methotrexate-Loaded Lipid-Polymer Hybrid Nanoparticles for Controlled Drug Delivery Applications. Int. J. Pharm..

[21] Zhao P., Wang H., Yu M., Liao Z., Wang X., Zhang F., Ji W., Wu B., Han J., Zhang H. (2012). Paclitaxel Loaded Folic Acid Targeted Nanoparticles of Mixed Lipid-Shell and Polymer-Core: In Vitro and in Vivo Evaluation. Eur. J. Pharm. Biopharm..

[22] Almeida A.J., Runge S., Müller R.H. (1997). Peptide-Loaded Solid Lipid Nanoparticles (SLN): Influence of Production Parameters. Int. J. Pharm..

[23] Garcıa-Fuentes M., Torres D., Alonso M.J. (2003). Design of Lipid Nanoparticles for the Oral Delivery of Hydrophilic Macromolecules. Colloids Surf. B Biointerfaces.

[24] Liu Y., Li K., Pan J., Liu B., Feng S.-S. (2010). Folic Acid Conjugated Nanoparticles of Mixed Lipid Monolayer Shell and Biodegradable Polymer Core for Targeted Delivery of Docetaxel. Biomaterials.

[25] Seedat N., Kalhapure R.S., Mocktar C., Vepuri S., Jadhav M., Soliman M., Govender T. (2016). Co-Encapsulation of Multi-Lipids and Polymers Enhances the Performance of Vancomycin in Lipid–Polymer Hybrid Nanoparticles: In Vitro and in Silico Studies. Mater. Sci. Eng. C.

[26] Zheng Y., Yu B., Weecharangsan W., Piao L., Darby M., Mao Y., Koynova R., Yang X., Li H., Xu S. (2010). Transferrin-Conjugated Lipid-Coated PLGA Nanoparticles for Targeted Delivery of Aromatase Inhibitor 7α-APTADD to Breast Cancer Cells. Int. J. Pharm..

[27] Kamaly N., Yameen B., Wu J., Farokhzad O.C. (2016). Degradable Controlled-Release Polymers and Polymeric Nanoparticles: Mechanisms of Controlling Drug Release. Chem. Rev..

[28] Mieszawska A.J., Gianella A., Cormode D.P., Zhao Y., Meijerink A., Langer R., Farokhzad O.C., Fayad Z.A., Mulder W.J.M. (2012). Engineering of Lipid-Coated PLGA Nanoparticles with a Tunable Payload of Diagnostically Active Nanocrystals for Medical Imaging. Chem. Commun..

[29] Le L., Bokare A., Erogbogbo F. (2018). Hand Powered, Cost Effective, 3D Printed Nanoparticle Synthesizer: Effects of Polymer End Caps, Drugs, and Solvents on Lipid Polymer Hybrid Nanoparticles. Mater. Res. Express.

[30] Alsaab H.O., Alharbi F.D., Alhibs A.S., Alanazi N.B., Alshehri B.Y., Saleh M.A., Alshehri F.S., Algarni M.A., Almugaiteeb T., Uddin M.N. (2022). PLGA-Based Nanomedicine: History of Advancement and Development in Clinical Applications of Multiple Diseases. Pharmaceutics.

[31] Muddineti O.S., Omri A. (2022). Current Trends in PLGA Based Long-Acting Injectable Products: The Industry Perspective. Expert Opin. Drug Deliv..

[32] Khouri N.G., Bahú J.O., Blanco-Llamero C., Severino P., Concha V.O.C., Souto E.B. (2024). Polylactic Acid (PLA): Properties, Synthesis, and Biomedical Applications—A Review of the Literature. J. Mol. Struct..

[33] Espinoza S.M., Patil H.I., San Martin Martinez E., Casañas Pimentel R., Ige P.P. (2020). Poly-ε-Caprolactone (PCL), a Promising Polymer for Pharmaceutical and Biomedical Applications: Focus on Nanomedicine in Cancer. Int. J. Polym. Mater. Polym. Biomater..

[34] Tan S., Sasada T., Bershteyn A., Yang K., Ioji T., Zhang Z. (2014). Combinational Delivery of Lipid-Enveloped Polymeric Nanoparticles Carrying Different Peptides for Anti-Tumor Immunotherapy. Nanomedicine.

[35] Khan M.M., Madni A., Torchilin V., Filipczak N., Pan J., Tahir N., Shah H. (2019). Lipid-Chitosan Hybrid Nanoparticles for Controlled Delivery of Cisplatin. Drug Deliv..

[36] Bhardwaj A., Mehta S., Yadav S., Singh S.K., Grobler A., Goyal A.K., Mehta A. (2016). Pulmonary Delivery of Antitubercular Drugs Using Spray-Dried Lipid–Polymer Hybrid Nanoparticles. Artif. Cells Nanomed. Biotechnol..

[37] Thevenot J., Troutier A.-L., David L., Delair T., Ladavière C. (2007). Steric Stabilization of Lipid/Polymer Particle Assemblies by Poly(Ethylene Glycol)-Lipids. Biomacromolecules.

[38] Gajbhiye K.R., Salve R., Narwade M., Sheikh A., Kesharwani P., Gajbhiye V. (2023). Lipid Polymer Hybrid Nanoparticles: A Custom-Tailored next-Generation Approach for Cancer Therapeutics. Mol. Cancer.

[39] Boushra M., Tous S., Fetih G., Xue H.-Y., Tran N.T., Wong H.L. (2016). Methocel-Lipid Hybrid Nanocarrier for Efficient Oral Insulin Delivery. J. Pharm. Sci..

[40] Grigoras A.G. (2017). Polymer-Lipid Hybrid Systems Used as Carriers for Insulin Delivery. Nanomed. Nanotechnol. Biol. Med..

[41] Cui F., Shi K., Zhang L., Tao A., Kawashima Y. (2006). Biodegradable Nanoparticles Loaded with Insulin–Phospholipid Complex for Oral Delivery: Preparation, in Vitro Characterization and in Vivo Evaluation. J. Control. Release.

[42] Karlsson J., Rhodes K.R., Green J.J., Tzeng S.Y. (2020). Poly(Beta-Amino Ester)s as Gene Delivery Vehicles: Challenges and Opportunities. Expert Opin. Drug Deliv..

[43] Zhang H., Dong S., Zhang S., Li Y., Li J., Dai Y., Wang D. (2021). pH-Responsive Lipid Polymer Hybrid Nanoparticles (LPHNs) Based on Poly (β-Amino Ester) as a Promising Candidate to Resist Breast Cancers. J. Drug Deliv. Sci. Technol..

[44] Khodaei M., Rostamizadeh K., Taromchi A.H., Monirinasab H., Fathi M. (2021). DDAB Cationic Lipid-mPEG, PCL Copolymer Hybrid Nano-Carrier Synthesis and Application for Delivery of siRNA Targeting IGF-1R into Breast Cancer Cells. Clin. Transl. Oncol..

[45] Li L., He X., Su H., Zhou D., Song H., Wang L., Jiang X. (2015). Poly(Ethylene Glycol)-Block-Poly(ε-Caprolactone—and Phospholipid-Based Stealth Nanoparticles with Enhanced Therapeutic Efficacy on Murine Breast Cancer by Improved Intracellular Drug Delivery. IJN.

[46] Wang C., Sui X., Simbar N., Sjollema K.A., van Hest J.C., Zuhorn I.S. (2016). Hybrid Nanoparticles Composed of pH-Sensitive PDPA-b-PCL-b-PEG Copolymer and DOTAP Cationic Lipid Show a Two-Step Mechanism of Endosomal Escape. Biomimetic Drug Nanocarriers to Overcome Biological Barriers: Inspiration from Pathogen Invasion.

[47] Liu J.-L., Li J., Zhang L.-Y., Zhang P.-L., Zhou J.-L., Liu B. (2017). Preparation of N, N, N-Trimethyl Chitosan-Functionalized Retinoic Acid-Loaded Lipid Nanoparticles for Enhanced Drug Delivery to Glioblastoma. Trop. J. Pharm. Res..

[48] Santos J.C.C., Moreno P.M.D., Mansur A.A.P., Leiro V., Mansur H.S., Pêgo A.P. (2015). Functionalized Chitosan Derivatives as Nonviral Vectors: Physicochemical Properties of Acylated N,N,N-Trimethyl Chitosan/Oligonucleotide Nanopolyplexes. Soft Matter.

[49] Massadeh S., Omer M.E., Alterawi A., Ali R., Alanazi F.H., Almutairi F., Almotairi W., Alobaidi F.F., Alhelal K., Almutairi M.S. (2020). Optimized Polyethylene Glycolylated Polymer–Lipid Hybrid Nanoparticles as a Potential Breast Cancer Treatment. Pharmaceutics.

[50] Mutlu-Agardan N.B., Han S. (2021). In Vitro and in Vivo Evaluations on Nanoparticle and Phospholipid Hybrid Nanoparticles with Absorption Enhancers for Oral Insulin Delivery. Pharm. Dev. Technol..

[51] Ling G., Zhang P., Zhang W., Sun J., Meng X., Qin Y., Deng Y., He Z. (2010). Development of Novel Self-Assembled DS-PLGA Hybrid Nanoparticles for Improving Oral Bioavailability of Vincristine Sulfate by P-Gp Inhibition. J. Control. Release.

[52] Qin L., Wu H., Xu E., Zhang X., Guan J., Zhao R., Mao S. (2021). Exploring the Potential of Functional Polymer-Lipid Hybrid Nanoparticles for Enhanced Oral Delivery of Paclitaxel. Asian J. Pharm. Sci..

[53] Feng Q., Zhang L., Liu C., Li X., Hu G., Sun J., Jiang X. (2015). Microfluidic Based High Throughput Synthesis of Lipid-Polymer Hybrid Nanoparticles with Tunable Diameters. Biomicrofluidics.

[54] González-García D., Tapia O., Évora C., García-García P., Delgado A. (2025). Conventional and Microfluidic Methods: Design and Optimization of Lipid-Polymeric Hybrid Nanoparticles for Gene Therapy. Drug Deliv. Transl. Res..

[55] Boushra M., Tous S., Fetih G., Xue H.-Y., Wong H.-L. (2019). Development of Bi-Polymer Lipid Hybrid Nanocarrier (BLN) to Improve the Entrapment and Stability of Insulin for Efficient Oral Delivery. J. Drug Deliv. Sci. Technol..

[56] Hu C.-M.J., Kaushal S., Cao H.S.T., Aryal S., Sartor M., Esener S., Bouvet M., Zhang L. (2010). Half-Antibody Functionalized Lipid−Polymer Hybrid Nanoparticles for Targeted Drug Delivery to Carcinoembryonic Antigen Presenting Pancreatic Cancer Cells. Mol. Pharm..

[57] Valencia P.M., Basto P.A., Zhang L., Rhee M., Langer R., Farokhzad O.C., Karnik R. (2010). Single-Step Assembly of Homogenous Lipid−Polymeric and Lipid−Quantum Dot Nanoparticles Enabled by Microfluidic Rapid Mixing. ACS Nano.

[58] Parveen S., Gupta P., Kumar S., Banerjee M. (2023). Lipid Polymer Hybrid Nanoparticles as Potent Vehicles for Drug Delivery in Cancer Therapeutics. Med. Drug Discov..

[59] Kassaee S.N., Richard D., Ayoko G.A., Islam N. (2024). Lipid Polymer Hybrid Nanoparticles against Lung Cancer and Their Application as Inhalable Formulation. Nanomedicine.

[60] Pang J., Xing H., Sun Y., Feng S., Wang S. (2020). Non-Small Cell Lung Cancer Combination Therapy: Hyaluronic Acid Modified, Epidermal Growth Factor Receptor Targeted, pH Sensitive Lipid-Polymer Hybrid Nanoparticles for the Delivery of Erlotinib plus Bevacizumab. Biomed. Pharmacother..

[61] Guo P., Xue H.Y., Buttaro B.A., Tran N.T., Wong H.L. (2020). Enhanced Eradication of Intracellular and Biofilm-Residing Methicillin-Resistant Staphylococcus Aureus (MRSA) Reservoirs with Hybrid Nanoparticles Delivering Rifampicin. Int. J. Pharm..

[62] Khan M.M., Madni A., Tahir N., Parveen F., Khan S., Jan N., Ali A., Abdurrahim M., Farooq U., Khan M.I. (2020). Co-Delivery of Curcumin and Cisplatin to Enhance Cytotoxicity of Cisplatin Using Lipid-Chitosan Hybrid Nanoparticles. IJN.

[63] Tahir N., Madni A., Li W., Correia A., Khan M.M., Rahim M.A., Santos H.A. (2020). Microfluidic Fabrication and Characterization of Sorafenib-Loaded Lipid-Polymer Hybrid Nanoparticles for Controlled Drug Delivery. Int. J. Pharm..

[64] Bokare A., Takami A., Kim J.H., Dong A., Chen A., Valerio R., Gunn S., Erogbogbo F. (2019). Herringbone-Patterned 3D-Printed Devices as Alternatives to Microfluidics for Reproducible Production of Lipid Polymer Hybrid Nanoparticles. ACS Omega.

[65] Sawant R.M., Hurley J.P., Salmaso S., Kale A., Tolcheva E., Levchenko T.S., Torchilin V.P. (2006). “SMART” Drug Delivery Systems: Double-Targeted pH-Responsive Pharmaceutical Nanocarriers. Bioconj. Chem..

[66] Mehta M., Bui T.A., Care A., Deng W. (2024). Targeted Polymer Lipid Hybrid Nanoparticles for In-Vitro siRNA Therapy in Triple-Negative Breast Cancer. J. Drug Deliv. Sci. Technol..

[67] Hu Y., Hoerle R., Ehrich M., Zhang C. (2015). Engineering the Lipid Layer of Lipid–PLGA Hybrid Nanoparticles for Enhanced in Vitro Cellular Uptake and Improved Stability. Acta Biomater..

[68] Mukherjee A., Waters A.K., Kalyan P., Achrol A.S., Kesari S., Yenugonda V.M. (2019). Lipid–Polymer Hybrid Nanoparticles as a next-Generation Drug Delivery Platform: State of the Art, Emerging Technologies, and Perspectives. IJN.

[69] Ferreira Soares D.C., Domingues S.C., Viana D.B., Tebaldi M.L. (2020). Polymer-Hybrid Nanoparticles: Current Advances in Biomedical Applications. Biomed. Pharmacother..

[70] Ramasamy T., Tran T.H., Choi J.Y., Cho H.J., Kim J.H., Yong C.S., Choi H.-G., Kim J.O. (2014). Layer-by-Layer Coated Lipid–Polymer Hybrid Nanoparticles Designed for Use in Anticancer Drug Delivery. Carbohydr. Polym..

[71] Elder D. (2017). ICH Q6A Specifications: Test Procedures and Acceptance Criteria for New Drug Substances and New Drug Products: Chemical Substances. ICH Qual. Guidel. Implement. Guide.

[72] European Medicines Agency (2011). ICH Development and Manufacture of Drug Substances (Chemical Entities and Biotechnological/Biological Entities) Q11.

[73] Hallan S.S., Kaur P., Kaur V., Mishra N., Vaidya B. (2016). Lipid Polymer Hybrid as Emerging Tool in Nanocarriers for Oral Drug Delivery. Artif. Cells Nanomed. Biotechnol..

[74] Liu Y., Xie X., Chen H., Hou X., He Y., Shen J., Shi J., Feng N. (2020). Advances in Next-Generation Lipid-Polymer Hybrid Nanocarriers with Emphasis on Polymer-Modified Functional Liposomes and Cell-Based-Biomimetic Nanocarriers for Active Ingredients and Fractions from Chinese Medicine Delivery. Nanomed. Nanotechnol. Biol. Med..

[75] Bangera P.D., Kara D.D., Tanvi K., Tippavajhala V.K., Rathnanand M. (2023). Highlights on Cell-Penetrating Peptides and Polymer-Lipid Hybrid Nanoparticle: Overview and Therapeutic Applications for Targeted Anticancer Therapy. AAPS PharmSciTech.

[76] Rahman M., Alharbi K.S., Alruwaili N.K., Anfinan N., Almalki W.H., Padhy I., Sambamoorthy U., Swain S., Beg S. (2020). Nucleic Acid-Loaded Lipid-Polymer Nanohybrids as Novel Nanotherapeutics in Anticancer Therapy. Expert Opin. Drug Deliv..

[77] Ruttala H.B., Ramasamy T., Ruttala R.R.T., Tran T.H., Jeong J.-H., Choi H.-G., Ku S.K., Yong C.S., Kim J.O. (2021). Mitochondria-Targeting Multi-Metallic ZnCuO Nanoparticles and IR780 for Efficient Photodynamic and Photothermal Cancer Treatments. J. Mater. Sci. Technol..

[78] Cheow W.S., Hadinoto K. (2011). Factors Affecting Drug Encapsulation and Stability of Lipid–Polymer Hybrid Nanoparticles. Colloids Surf. B Biointerfaces.

[79] Mandal B., Mittal N.K., Balabathula P., Thoma L.A., Wood G.C. (2016). Development and in Vitro Evaluation of Core–Shell Type Lipid–Polymer Hybrid Nanoparticles for the Delivery of Erlotinib in Non-Small Cell Lung Cancer. Eur. J. Pharm. Sci..

[80] Gameiro M., Mano J.F., Gaspar V.M. (2024). Emerging Lipid–Polymer Hybrid Nanoparticles for Genome Editing. Polym. Chem..

[81] Almawash S. (2025). Oral Bioavailability Enhancement of Anti-Cancer Drugs Through Lipid Polymer Hybrid Nanoparticles. Pharmaceutics.

[82] Subramanian D.A., Langer R., Traverso G. (2022). Mucus Interaction to Improve Gastrointestinal Retention and Pharmacokinetics of Orally Administered Nano-Drug Delivery Systems. J. Nanobiotechnol..

[83] Imam S.S., Gilani S.J., Bin Jumah M.N., Rizwanullah M., Zafar A., Ahmed M.M., Alshehri S. (2022). Harnessing Lipid Polymer Hybrid Nanoparticles for Enhanced Oral Bioavailability of Thymoquinone: In Vitro and In Vivo Assessments. Polymers.

[84] Cheng H., Cui Z., Guo S., Zhang X., Huo Y., Mao S. (2021). Mucoadhesive versus Mucopenetrating Nanoparticles for Oral Delivery of Insulin. Acta Biomater..

[85] Lemmer H.J.R., Hamman J.H. (2013). Paracellular Drug Absorption Enhancement through Tight Junction Modulation. Expert Opin. Drug Deliv..

[86] Jansen M.A.A., Klausen L.H., Thanki K., Lyngsø J., Skov Pedersen J., Franzyk H., Nielsen H.M., van Eden W., Dong M., Broere F. (2019). Lipidoid-Polymer Hybrid Nanoparticles Loaded with TNF siRNA Suppress Inflammation after Intra-Articular Administration in a Murine Experimental Arthritis Model. Eur. J. Pharm. Biopharm..

[87] Wang J. (2020). Combination Treatment of Cervical Cancer Using Folate-Decorated, pH-Sensitive, Carboplatin and Paclitaxel Co-Loaded Lipid-Polymer Hybrid Nanoparticles. DDDT.

[88] Prajapati J.B., Katariya H., Patel R. (2018). Peyer’e Patch Targeting of Isradipine Loaded Solid Lipid Nanoparticles: It’s Cellular Uptake Study. J. Drug Deliv. Sci. Technol..

[89] Patel M., Desai A., Kansara V., Vyas B. (2023). Core Shell Lipid-Polymer Hybrid Nanoparticles for Oral Bioavailability Enhancement of Ibrutinib via Lymphatic Uptake. AAPS PharmSciTech.

[90] He Y., Cheng M., Yang R., Li H., Lu Z., Jin Y., Feng J., Tu L. (2023). Research Progress on the Mechanism of Nanoparticles Crossing the Intestinal Epithelial Cell Membrane. Pharmaceutics.

[91] Bose R.J., Arai Y., Ahn J.C., Park H., Lee S.-H. (2015). Influence of Cationic Lipid Concentration on Properties of Lipid–Polymer Hybrid Nanospheres for Gene Delivery. IJN.

[92] Zhou X., Liu Y., Wang X., Li X., Xiao B. (2020). Effect of Particle Size on the Cellular Uptake and Anti-Inflammatory Activity of Oral Nanotherapeutics. Colloids Surf. B Biointerfaces.

[93] Jain S., Kumar M., Kumar P., Verma J., Rosenholm J.M., Bansal K.K., Vaidya A. (2023). Lipid–Polymer Hybrid Nanosystems: A Rational Fusion for Advanced Therapeutic Delivery. J. Funct. Biomater..

[94] Raman S., Mahmood S., Rahman A. (2020). A Review on Lipid-Polymer Hybrid Nanoparticles and Preparation with Recent Update. MSF.

[95] Zhang L., Zhang L. (2010). Lipid–Polymer Hybrid Nanoparticles: Synthesis, Characterization and Applications. Nano LIFE.

[96] Yang G., Liu Y., Jin S., Hui Y., Wang X., Xu L., Chen D., Weitz D., Zhao C.-X. (2023). Phase Separation-Induced Nanoprecipitation for Making Polymer Nanoparticles with High Drug Loading. Aggregate.

[97] Khalili L., Dehghan G., Sheibani N., Khataee A. (2022). Smart Active-Targeting of Lipid-Polymer Hybrid Nanoparticles for Therapeutic Applications: Recent Advances and Challenges. Int. J. Biol. Macromol..

[98] Craparo E.F., Cabibbo M., Scialabba C., Giammona G., Cavallaro G. (2022). Inhalable Formulation Based on Lipid–Polymer Hybrid Nanoparticles for the Macrophage Targeted Delivery of Roflumilast. Biomacromolecules.

[99] Meyer R.A., Hussmann G.P., Peterson N.C., Santos J.L., Tuesca A.D. (2022). A Scalable and Robust Cationic Lipid/Polymer Hybrid Nanoparticle Platform for mRNA Delivery. Int. J. Pharm..

[100] Shiraishi K., Kawano K., Maitani Y., Aoshi T., Ishii K.J., Sanada Y., Mochizuki S., Sakurai K., Yokoyama M. (2016). Exploring the Relationship between Anti-PEG IgM Behaviors and PEGylated Nanoparticles and Its Significance for Accelerated Blood Clearance. J. Control. Release.

[101] Dehaini D., Fang R.H., Luk B.T., Pang Z., Hu C.-M.J., Kroll A.V., Yu C.L., Gao W., Zhang L. (2016). Ultra-Small Lipid–Polymer Hybrid Nanoparticles for Tumor-Penetrating Drug Delivery. Nanoscale.

[102] Korucu Aktas P., Baysal I., Yabanoglu-Ciftci S., Arica B. (2023). Development and In Vitro Evaluation of Crizotinib-Loaded Lipid–Polymer Hybrid Nanoparticles Using Box–Behnken Design in Non-Small Cell Lung Cancer. AAPS PharmSciTech.

[103] Gusmão L.A., Tedesco A.C. (2022). Polymer–Lipid Hybrid Nanostructures for Drug Delivery. Hybrid Nanomaterials for Drug Delivery.

[104] Verma J., Singh N.K., Bansal K.K. (2024). Recent Patents in Polymer–Lipid Hybrid Nanoparticles Technology. Ther. Deliv..

[105] Zhang L., Chan J.M., Gu F.X., Rhee J.-W., Wang A.Z., Radovic-Moreno A.F., Alexis F., Langer R., Farokhzad O.C. (2008). Self-Assembled Lipid−Polymer Hybrid Nanoparticles: A Robust Drug Delivery Platform. ACS Nano.

[106] Hazari S.A., Sheikh A., Abourehab M.A.S., Tulbah A.S., Kesharwani P. (2023). Self-Assembled Gallic Acid Loaded Lecithin-Chitosan Hybrid Nanostructured Gel as a Potential Tool against Imiquimod-Induced Psoriasis. Environ. Res..

[107] Sun Y., Lee R.J., Meng F., Wang G., Zheng X., Dong S., Teng L. (2020). Microfluidic Self-Assembly of High Cabazitaxel Loading Albumin Nanoparticles. Nanoscale.

[108] Fabozzi A., Della Sala F., di Gennaro M., Barretta M., Longobardo G., Solimando N., Pagliuca M., Borzacchiello A. (2023). Design of Functional Nanoparticles by Microfluidic Platforms as Advanced Drug Delivery Systems for Cancer Therapy. Lab. A Chip.

[109] Bose R.J.C., Ravikumar R., Karuppagounder V., Bennet D., Rangasamy S., Thandavarayan R.A. (2017). Lipid–Polymer Hybrid Nanoparticle-Mediated Therapeutics Delivery: Advances and Challenges. Drug Discov. Today.

[110] Liu Z., Fontana F., Python A., Hirvonen J.T., Santos H.A. (2020). Microfluidics for Production of Particles: Mechanism, Methodology, and Applications. Small.

[111] Kim Y., Lee Chung B., Ma M., Mulder W.J.M., Fayad Z.A., Farokhzad O.C., Langer R. (2012). Mass Production and Size Control of Lipid–Polymer Hybrid Nanoparticles through Controlled Microvortices. Nano Lett..

[112] Li S., Yang B., Ye L., Hu S., Li B., Yang Y., Jia X., Feng L., Xiong Z. (2024). Timescale Dependent Homogeneous Assembly of Lipid-Polymer Hybrid Nanoparticles in Laminar Mixing for Enhanced Lung Cancer Treatment. Chem. Eng. J..

[113] Mandal B., Bhattacharjee H., Mittal N., Sah H., Balabathula P., Thoma L.A., Wood G.C. (2013). Core–Shell-Type Lipid–Polymer Hybrid Nanoparticles as a Drug Delivery Platform. Nanomed. Nanotechnol. Biol. Med..

[114] Devrim B., Kara A., Vural İ., Bozkır A. (2016). Lysozyme-Loaded Lipid-Polymer Hybrid Nanoparticles: Preparation, Characterization and Colloidal Stability Evaluation. Drug Dev. Ind. Pharm..

[115] Garcia-Fuentes M., Alonso M.J., Torres D. (2005). Design and Characterization of a New Drug Nanocarrier Made from Solid–Liquid Lipid Mixtures. J. Colloid Interface Sci..

[116] Shafique M., Ur Rehman M., Kamal Z., Alzhrani R.M., Alshehri S., Alamri A.H., Bakkari M.A., Sabei F.Y., Safhi A.Y., Mohammed A.M. (2023). Formulation Development of Lipid Polymer Hybrid Nanoparticles of Doxorubicin and Its In-Vitro, in-Vivo and Computational Evaluation. Front. Pharmacol..

[117] Yalcin T.E., Ilbasmis-Tamer S., Takka S. (2020). Antitumor Activity of Gemcitabine Hydrochloride Loaded Lipid Polymer Hybrid Nanoparticles (LPHNs): In Vitro and in Vivo. Int. J. Pharm..

[118] Shi J., Xiao Z., Votruba A.R., Vilos C., Farokhzad O.C. (2011). Differentially Charged Hollow Core/Shell Lipid-Polymer-Lipid Hybrid Nanoparticles for Small Interfering RNA Delivery. Angew. Chem. Int. Ed..

[119] Gartziandia O., Herran E., Pedraz J.L., Carro E., Igartua M., Hernandez R.M. (2015). Chitosan Coated Nanostructured Lipid Carriers for Brain Delivery of Proteins by Intranasal Administration. Colloids Surf. B Biointerfaces.

[120] García-Díaz M., Foged C., Nielsen H.M. (2015). Improved Insulin Loading in Poly(Lactic-Co-Glycolic) Acid (PLGA) Nanoparticles upon Self-Assembly with Lipids. Int. J. Pharm..

[121] Asuman Bozkır B.D. (2014). Preparation and Characterization of Protein-Loaded Lipid-Polymer Hybrid Nanoparticles with Polycaprolactone as Polymeric Core Material. J. Biomol. Res. Ther..

[122] Srinivasan C., Katare Y.K., Muthukumaran T., Panda A.K. (2005). Effect of Additives on Encapsulation Efficiency, Stability and Bioactivity of Entrapped Lysozyme from Biodegradable Polymer Particles. J. Microencapsul..

[123] Zafar A., Yasir M., Panda D.S., Khalid M., Singh L., Quazi A.M. (2024). Development of Lipid Polymer Hybrid Nanoparticles of Abietic Acid: Optimization, In-Vitro and Preclinical Evaluation. AAPS PharmSciTech.

[124] Kasif M., Gupta R., Singh P.P., Bhardwaj P., Goyal R., Bansal K.K., Mahor A.K. (2024). Development of Biocompatible Lipid-Polymer Hybrid Nanoparticles for Enhanced Oral Absorption of Posaconazole: A Mechanistic in Vitro and in Silico Assessment. J. Drug Deliv. Sci. Technol..

[125] Asuman Bozkır B.D. (2015). Design and Evaluation of Hydrophobic Ion-Pairing Complexation of Lysozyme with Sodium Dodecyl Sulfate for Improved Encapsulation of Hydrophilic Peptides/Proteins by Lipid-Polymer Hybrid Nanoparticles. J. Nanomed. Nanotechnol..

[126] Sarmento B., Mazzaglia D., Bonferoni M.C., Neto A.P., do Céu Monteiro M., Seabra V. (2011). Effect of Chitosan Coating in Overcoming the Phagocytosis of Insulin Loaded Solid Lipid Nanoparticles by Mononuclear Phagocyte System. Carbohydr. Polym..

[127] Boushra M., Tous S., Fetih G., Korzekwa K., Lebo D.B., Xue H.Y., Wong H.L. (2016). Development and Evaluation of Viscosity-Enhanced Nanocarrier (VEN) for Oral Insulin Delivery. Int. J. Pharm..

[128] Ebrahimian M., Mahvelati F., Malaekeh-Nikouei B., Hashemi E., Oroojalian F., Hashemi M. (2022). Bromelain Loaded Lipid-Polymer Hybrid Nanoparticles for Oral Delivery: Formulation and Characterization. Appl. Biochem. Biotechnol..

[129] Hanson L.R., Frey W.H. (2008). Intranasal Delivery Bypasses the Blood-Brain Barrier to Target Therapeutic Agents to the Central Nervous System and Treat Neurodegenerative Disease. BMC Neurosci..

[130] Kumar M., Misra A., Mishra A.K., Mishra P., Pathak K. (2008). Mucoadhesive Nanoemulsion-Based Intranasal Drug Delivery System of Olanzapine for Brain Targeting. J. Drug Target..

[131] Ahsan S.M., Thomas M., Reddy K.K., Sooraparaju S.G., Asthana A., Bhatnagar I. (2018). Chitosan as Biomaterial in Drug Delivery and Tissue Engineering. Int. J. Biol. Macromol..

[132] Olah I., Lasher J., Regdon G., Pintye-Hodi K., Baki G., Sovany T. (2019). Evaluating Superdisintegrants for Their Performance in Orally Disintegrating Tablets Containing Lysozyme Enzyme. J. Drug Deliv. Sci. Technol..

[133] Oderinde B., Agbede O., Iheukwumere I., Ghamba P., Medugu J., Oku E. (2017). Antiviral Activity of Hen Egg-White Lysozyme on Polio Virus. Sokoto J. Med. Lab. Sci..

[134] Oliver W.T., Wells J.E. (2015). Lysozyme as an Alternative to Growth Promoting Antibiotics in Swine Production. J. Anim. Sci. Biotechnol..

[135] Borges O., Borchard G., Verhoef J.C., de Sousa A., Junginger H.E. (2005). Preparation of Coated Nanoparticles for a New Mucosal Vaccine Delivery System. Int. J. Pharm..

[136] Chesnut C.H., Azria M., Silverman S., Engelhardt M., Olson M., Mindeholm L. (2008). Salmon Calcitonin: A Review of Current and Future Therapeutic Indications. Osteoporos. Int..

[137] Plosker G.L., McTavish D. (1996). Intranasal Salcatonin (Salmon Calcitonin). Drugs Aging.

[138] Luo Y.Y., Xiong X.Y., Tian Y., Li Z.L., Gong Y.C., Li Y.P. (2016). A Review of Biodegradable Polymeric Systems for Oral Insulin Delivery. Drug Deliv..

[139] Németh Z., Pallagi E., Dobó D.G., Csóka I. (2020). A Proposed Methodology for a Risk Assessment-Based Liposome Development Process. Pharmaceutics.

[140] Giovino C., Ayensu I., Tetteh J., Boateng J.S. (2013). An Integrated Buccal Delivery System Combining Chitosan Films Impregnated with Peptide Loaded PEG-b-PLA Nanoparticles. Colloids Surf. B Biointerfaces.

[141] Hu S., Pei X., Duan L., Zhu Z., Liu Y., Chen J., Chen T., Ji P., Wan Q., Wang J. (2021). A Mussel-Inspired Film for Adhesion to Wet Buccal Tissue and Efficient Buccal Drug Delivery. Nat. Commun..

[142] Morales J.O., Huang S., Williams R.O., McConville J.T. (2014). Films Loaded with Insulin-Coated Nanoparticles (ICNP) as Potential Platforms for Peptide Buccal Delivery. Colloids Surf. B Biointerfaces.

[143] Morales J.O., Ross A.C., McConville J.T. (2013). Protein-Coated Nanoparticles Embedded in Films as Delivery Platforms. J. Pharm. Pharmacol..

[144] Mortazavian E., Dorkoosh F.A., Rafiee-Tehrani M. (2014). Design, Characterization and Ex Vivo Evaluation of Chitosan Film Integrating of Insulin Nanoparticles Composed of Thiolated Chitosan Derivative for Buccal Delivery of Insulin. Drug Dev. Ind. Pharm..

